# Potentiation of Penicillin G and Selected β-Lactams with Quercetin Against Multidrug-Resistant Bacteria: Mechanistic Insights, Antibacterial Phytochemicals, and Toxicity Evaluation

**DOI:** 10.3390/ijms27135825

**Published:** 2026-06-27

**Authors:** Gagan Tiwana, Ian Edwin Cock, Matthew James Cheesman

**Affiliations:** 1 School of Pharmacy and Medical Sciences, Griffith University, Gold Coast QLD 4222, Australia; gagan.tiwana@griffithuni.edu.au; 2 School of Environment and Science, Griffith University, Brisbane QLD 4111, Australia; i.cock@griffith.edu.au

**Keywords:** phytochemical, antibacterial, synergy, metabolomics, fractional inhibitory concentration, isobolograms, mechanism, toxicity

## Abstract

Antimicrobial resistance is increasing, necessitating the development of novel and efficacious therapies. Plants contain phytochemicals, some of which may possess antibacterial properties. This research employed broth dilution experiments to investigate the antibacterial efficacy of fifteen phytochemicals identified in medicinal plant extracts. The sum of fractional inhibitory concentration of phytochemicals in conjunction with reference antibiotics were also analysed. The inhibitory effects of phytochemicals against β-lactamase were evaluated to explore their potential mechanisms of action. The phytochemicals were evaluated for toxicity on human dermal fibroblast cells. Gallic acid and luteolin significantly inhibited *Staphylococcus aureus* and the methicillin-resistant *S. aureus* (MRSA) strain, with minimum inhibitory concentration (MICs) of 62.5 µg/mL. Gallic acid also demonstrated restricted efficacy against Gram-negative species, with MICs ranging from 312.5 to 1250 µg/mL. Gram-negative bacteria exhibited no response to luteolin. Ellagic acid, catechin, naringenin, and quercetin exhibited moderate antibacterial efficacy against the tested pathogens (625–2500 µg/mL MIC). Corilagin exhibited significant antibacterial activity against *S. aureus* and MRSA, with a MIC of 7.81 µg/mL. Corilagin also exhibited notable efficacy against *Bacillus cereus*, *Shigella flexneri*, and *Klebsiella pneumoniae*, with MICs ranging from 62.5 to 250 µg/mL. Fractional inhibitory concentration studies revealed a synergistic effect between amoxicillin and corilagin against *B. cereus*. Additionally, catechin, luteolin, and quercetin synergised penicillin G against *S. aureus*. Quercetin potentiated the activity of β-lactams (amoxicillin, penicillin G, and oxacillin) against MRSA. Notably, these antibiotics were ineffective against MRSA alone. Isobologram analysis revealed potentiation between penicillin G and quercetin against MRSA at all tested ratios. The β-lactamase inhibitory activity of the phytochemicals was evaluated using a commercial screening kit, and the percentage of relative inhibition was determined. Quercetin and luteolin both inhibited β-lactamase, achieving relative inhibition rates of 77–100% across two time intervals. All phytochemicals were nontoxic against human dermal fibroblasts. Indeed, quercetin enhanced cell survival by 200%.

## 1. Introduction

Recent increases in bacterial antimicrobial resistance have created a pressing need for new antibacterial agents. Plants harbour a plethora of structurally diverse phytochemicals with antimicrobial potential [1] and are therefore promising targets for antibiotic discovery. Amongst these, phenolics and flavonoids have been widely studied due to their multiple antibiotic mechanisms, including cell wall damage, enzyme inhibition, efflux pump interference, and synergy with conventional antibiotics [2,3].

Our previous studies conducted an initial liquid-chromatography mass spectroscopy (LC-MS) analysis of Ayurvedic medicinal plant extracts with therapeutic antibacterial potential [4,5,6,7,8], including *Terminalia bellirica* Gaertn. Roxb. (TB), *Terminalia chebula* Retz. (TCh), *Phyllanthus emblica* Linn. (EO), *Phyllanthus niruri* Linn. (PN), *Azadirachta indica* A. Juss. (AzI), *Ocimum tenuiflorum* Linn. (OT), and *Solanum nigrum* Linn. (SN). A total of 174 individual phytochemicals were selected from the LC-MS data and identified across the seven species (Supplementary Table S1). Table S1 presents the LC–MS profiling data of the medicinal plant extracts, comprising 174 putatively identified phytochemicals. These data provided the phytochemical framework for the selection of representative and biologically relevant compounds for subsequent antibacterial, antibiotic potentiation, β-lactamase inhibition, and cytotoxicity evaluations.

Of these, fifteen compounds, gallic acid, ellagic acid, naringenin, luteolin, corilagin, catechin hydrate, isorhamnetin, vitexin, hesperetin, rutin hydrate, kaempferol, quercetin, galangin, hyperoside, and icariside II were selected for detailed study. The selection was governed by several pragmatic and scientific considerations: (i) they were among the more abundant or consistently detected constituents across LC-MS profiles of the plant extracts; (ii) they are readily available commercially; (iii) they represent structurally diverse subclasses (simple phenolics, tannins, flavonols/flavones, glycosides) that may display distinct modes of action; (iv) their safety and cytotoxic profiles are partly documented in the literature, affording a baseline for comparison; and (v) they allow exploration of synergistic or antagonistic interactions (FIC/isobologram) when combined, particularly in the context of antibiotic adjuvancy.

Each of the fifteen compounds was subjected to broth microdilution assays to determine minimum inhibitory concentrations (MICs) against target bacterial strains. Where feasible, fractional inhibitory concentration (ƩFIC) index and isobologram analysis were used to assess combinatorial interaction classes (synergy, additivity, non-interactive, antagonism). To further elucidate modes of resistance modulation, β-lactamase’s inhibition capacity of selected individual phytochemicals was also examined. Finally, recognising that a promising antibacterial agent must also be safe to human tissues, we evaluated cytotoxicity on adult human dermal fibroblast (HDFsa) cells to determine therapeutic windows. Collectively, this approach aims to (i) identify potent single-compound antibacterial leads, (ii) reveal synergistic combinations that may lower effective doses or circumvent resistance, (iii) uncover enzyme inhibition as a mechanism of resistance reversal, and (iv) confirm biocompatibility in a relevant human cell model. In so doing, this work helps bridge the gap between broad LC-MS phytochemical profiling and mechanistic, translational antibiotic development.

Previous studies have demonstrated that certain plant-derived phytochemicals, including flavonoids, possess intrinsic antibacterial activity and can potentiate the activity of conventional antibiotics, including β-lactams [2,3]. Furthermore, enzyme inhibitory activity and cytotoxicity profiles of several individual phytochemicals have also been reported. However, comprehensive studies integrating these biological evaluations using phytochemicals selected through LC-MS profiling of medicinal plant extracts against both susceptible and resistant clinically relevant bacterial pathogens remain limited.

Based on phytochemicals identified through LC–MS analysis of medicinal plant extracts, we hypothesised that selected phytochemicals may possess antibacterial activity against both susceptible and resistant bacterial pathogens and enhance the efficacy of conventional antibiotics. In this study, selected phytochemicals were comprehensively evaluated for their antibacterial activity against clinically relevant susceptible and resistant bacterial strains, including *Staphylococcus aureus*, methicillin-resistant *S. aureus* (MRSA), *Escherichia coli*, extended-spectrum β-lactamase (ESBL)-producing *E. coli*, *Klebsiella pneumoniae*, ESBL-producing *K. pneumoniae*, *Bacillus cereus*, *Shigella sonnei*, *Shigella flexneri*, and *Salmonella typhimurium*. Accordingly, the aims of this study were to evaluate the antibacterial activity of selected phytochemicals, investigate phytochemical–antibiotic interactions using fractional inhibitory concentration (FIC) and isobologram analyses, assess β-lactamase inhibitory activity as a potential mechanism underlying antibacterial enhancement, and determine cytotoxicity against adult human dermal fibroblast (HDFa) cells.

## 2. Results

### 2.1. Antibacterial Analysis

Table 1 depicts the MIC values (expressed as µM) of the phytochemicals against the bacterial pathogens. Corilagin was the most potent phytochemical across the all bacteria tested, inhibiting *S. aureus* and MRSA at 7.81 µg/mL (12.31 µM), *B. cereus* at 62.5 µg/mL (98.47 µM), *S. flexneri* at 125 µg/mL (196.93 µM), and *K. pneumoniae* at 250 µg/mL (393.86 µM). In contrast, corilagin was inactive against ESBL *K. pneumoniae*, *E. coli*, ESBL *E. coli*, *S. sonnei*, and *S. typhimurium*.

Gallic acid demonstrated broad albeit moderate-spectrum activity. It was strongly active against *S. aureus* and MRSA with an MIC of 62.5 µg/mL (367.56 µM). Moderate inhibition was observed against *K. pneumoniae* (312.5 µg/mL; 1837.15 µM), ESBL *K. pneumoniae* (625 µg/mL; 3674.3 µM), *B. cereus* (625 µg/mL; 3674.3 µM), *S. flexneri* and *S. typhimurium* (625 µg/mL; 3674.3 µM), and weak inhibition against *S. sonnei*, *E. coli* and ESBL *E. coli* at 1250 µg/mL (7348.6 µM).

Luteolin exhibited selective activity, being effective against *S. aureus* and MRSA at 62.5 µg/mL (218.38 µM), with no measurable inhibition of other bacterial strains at any concentration tested. Catechin showed moderate inhibition against *S. aureus* and MRSA with MIC values of 625 µg/mL (2026.5 µM), but was less active against *B. cereus*, *S. flexneri*, *S. sonnei*, and *S. typhimurium*, with MICs of 1250 µg/mL (4053 µM). Naringenin was moderately active against *S. aureus* and MRSA with an MIC of 625 µg/mL (2295.07 µM) and showed similar activity against *B. cereus* at the same concentration. No effect was observed on the remaining bacterial species. Ellagic acid inhibited *S. aureus* at 625 µg/mL (2068.41 µM), although it lacked activity against MRSA. It also displayed a weak inhibition of *S. flexneri* and *S. typhimurium* at 625 µg/mL (2068.41 µM) and *S. sonnei* at 1250 µg/mL (4136.82 µM).

Quercetin required high concentrations to inhibit growth, with MICs of 1250 µg/mL (2068.23 µM) against *S. aureus* and 2500 µg/mL (8272.92 µM) against MRSA, 625 µg/mL (2068.23 µM) against *S. flexneri* and *S. typhimurium*, and 1250 µg/mL (4136.46 µM) against *S. sonnei.* The remaining compounds were inactive across all strains tested.

To provide a reference framework, the MICs of commonly used antibiotics representing different drug classes were assessed against the same bacterial panel. β-Lactams (amoxicillin, cefazolin, ticarcillin, ceftriaxone, oxacillin, dicloxacillin, penicillin G) displayed variable activity against those bacteria. Dicloxacillin were highly effective against *S. aureus* with an MIC of 0.16 µg/mL (0.34 µM), but less effective against MRSA with an MIC of 2.5 µg/mL (5.32 µM). Penicillin G and amoxicillin were moderately active against *S. aureus* (1.25 µg/mL; equivalent to 3.74 µM and 3.42 µM, respectively), but were ineffective against MRSA. Amoxicillin were substantially weaker against Gram-negative species, requiring 2.5 µg/mL (6.84 µM). Cefazolin retained strong activity against *S. aureus* (0.63 µg/mL; 1.39 µM), but was inactive against MRSA. Also, cefazolin was less effective against Enterobacteriaceae (1.25–2.5 µg/mL; 2.75–5.5 µM). Ceftriaxone stood out as the most potent β-lactam for Gram-negative bacteria, inhibiting *E. coli*, *K. pneumoniae*, *S. flexneri*, *S. sonnei*, and *S. typhimurium* at 0.16–0.31 µg/mL (0.29–0.56 µM), although higher concentrations (2.5 µg/mL; 4.51 µM) were required to inhibit *S. aureus* growth. Ticarcillin was a weak antibacterial agent, requiring 2.5 µg/mL (6.50 µM) for inhibition of Gram-negative strains and showing no activity against *S. aureus.*

Aminoglycosides (gentamicin) showed selective activity. Gentamicin inhibited *K. pneumoniae* and *B. cereus* with an MIC of 0.31 µg/mL (0.65 µM), but was less effective against *E. coli*, *S. flexneri*, and *S. sonnei* (1.25–2.5 µg/mL; 2.62–5.23 µM), and inactive against *S. aureus*, MRSA, and ESBL *E. coli.* The macrolide antibiotic erythromycin was effective against Gram-positive bacteria. Indeed, erythromycin inhibited *S. aureus* at 0.31 µg/mL (0.42 µM) and *B. cereus* at 0.16 µg/mL (0.22 µM) but had no measurable effect on Gram-negative strains at any concentration tested.

Tetracycline had broad and potent antibacterial activity. Indeed, it was one of the most effective drug classes tested herein. MICs were as low as 0.02 µg/mL (0.05 µM) for *B. cereus*, 0.04 µg/mL (0.09 µM) for MRSA, and 0.08 µg/mL (0.18 µM) for *S. aureus*, whilst Gram-negative strains were inhibited at 0.31 µg/mL (0.70 µM). This consistent inhibition at low concentration across diverse pathogens highlights tetracycline’s broad-spectrum and reliability. Chloramphenicol demonstrated only weak activity, requiring 1.25–2.5 µg/mL (3.87–7.74 µM) to inhibit Gram-negative strains and showing no effect against *S. aureus* or MRSA. The glycopeptide vancomycin had moderate potency against Gram-positive bacteria, with MICs of 1.25 µg/mL (0.86 µM) for *S. aureus* and 0.63 µg/mL (0.43 µM) for MRSA and *B. cereus*, although chloramphenicol was inactive against Gram-negative organisms.

Ciprofloxacin was consistently the most potent agent. Ciprofloxacin inhibited Gram-negative strains at MIC of 0.02 µg/mL (0.06 µM) and remained active against *B. cereus* [0.16 µg/mL; (0.48 µM)], *S. aureus* and MRSA [0.63 µg/mL; (1.90 µM)]. Notably, it was the only antibiotic to show activity against ESBL *K. pneumoniae* [0.63 µg/mL; (1.90 µM)]. Polymyxin B showed strong selective activity against Enterobacteriaceae, with MICs of 0.02 µg/mL (0.08 µM) for *S. flexneri*, *S. sonnei*, and *S. typhimurium*, but 1.25–2.5 µg/mL (1.04–2.08 µM) for *E. coli* and *K. pneumoniae.* In contrast, polymyxin B was ineffective against *S. aureus*, MRSA and *B. cereus* at all concentrations tested.

### 2.2. Genomic Sequencing of ESBL Escherichia Coli Clinical Strain

Whole genome sequencing was conducted on the ESBL-producing E. coli isolate because to its significant multidrug resistance, especially against β-lactam antibiotics, compared to all other examined isolates. The sequencing was performed to uncover the genetic factors responsible for its broad resistance profile, including genes linked to ESBL formation and other antimicrobial resistance pathways. Furthermore, whole genome analysis elucidated the molecular foundation of resistance and the possible existence of virulence-associated genes. Whole-genome analysis of the ESBL *E. coli* isolate using CARD, NCBI, and ResFinder revealed a diverse repertoire of antimicrobial resistance determinants. All three databases consistently identified extended-spectrum β-lactamase (ESBL) genes, including *blaCTX-M-64* and *blaTEM-1*, as well as *blaEC-8*, confirming the ESBL ioslate. The results of the NCBI and ResFinder sequence identifications are shown in Table 2. The CARD analysis outcomes, containing a more detailed description of the findings, are shown in Supplementary Table S2.

Multiple aminoglycoside resistance determinants were present (aph(3′)-IIa, aph(6)-Id, aph(3″)-Ib, aadA2, rmtB), with rmtB conferring high-level aminoglycoside resistance. Resistance to sulphonamides and trimethoprim (sul1, sul2, dfrA12), tetracyclines (tetA), macrolides (mphA), and phenicols (floR) was also confirmed across all three databases. In addition, CARD identified a broad set of efflux pumps and global regulators (e.g., acrAB-TolC, oqxAB, emr family, mdt family, marA, cpxA, evgS/A, H-NS), which likely enhance tolerance to multiple antibiotic classes. CARD further detected the genes bacA, ugd, PmrF, and eptA, which are associated with cell envelope modification and potential polymyxin resistance pathways.

Phenotypic antibiotic susceptibility testing supported these genomic findings. The isolate demonstrated resistance to all tested agents, including β-lactams (penicillin G, oxacillin, dicloxacillin, amoxicillin, ticarcillin, cefazolin, ceftriaxone), fluoroquinolone (ciprofloxacin), macrolide (erythromycin), tetracycline, phenicol (chloramphenicol), aminoglycoside (gentamicin), and glycopeptide (vancomycin). Notably, only polymyxin B remained active at 2.5 µg/mL but this is still relatively high for pure antibiotic and is consistent with resistance. Taken together, these results reveal that this ESBL E. coli strain possesses an extensive genetic arsenal, conferring multidrug resistance across nearly all clinically relevant antibiotic classes. The concordance between genomic predictions and phenotypic resistance patterns emphasises the clinical challenge posed by this isolate, where polymyxin B represents the only remaining effective (albeit weak) therapeutic option.

### 2.3. Fractional Inhibitory Concentration (FIC) Analysis

A total of 224 phytochemical–antibiotic combinations were tested against the selected bacterial strains. Analysis of the FIC indices (Table 3) showed that seven combinations (3.1%) were synergistic (FIC ≤ 0.5), whilst 19 combinations (8.5%) exhibited additive effects (0.5 < FIC ≤ 1.0). The majority, comprising 188 combinations (83.9%), fell within the indifferent range (1.0 < FIC ≤ 4.0), indicating little or no enhancement of antibacterial activity when the agents were combined. Only 10 combinations (4.5%) demonstrated antagonism (FIC > 4.0), highlighting combinations that should be avoided therapeutically.

Strain-specific patterns were observed. For *B. cereus*, synergy was identified in corilagin–amoxicillin combinations, with an FIC of 0.19, whilst combinations of catechin–amoxicillin, and naringenin–gentamicin showed additive outcomes. The combinations of gallic acid with amoxicillin, chloramphenicol, cefazolin, and gentamicin exhibited additive interactions against *Shigella sonnei.* Similarly, the quercetin–gentamicin combination also showed an additive effect against the same strain. All combinations showed indifferent effects when tested against *S. flexneri.* Antagonistic interactions were observed for the combinations of catechin–ceftriaxone and gallic acid–gentamicin when tested against *S. typhimurium.* Quercetin also showed antagonistic effects when combined with amoxicillin, ceftriaxone, cefazolin, and gentamicin against the same strain.

Synergistic interactions were observed against *S. aureus* when penicillin G was combined with catechin, luteolin, or quercetin, yielding FIC values of 0.19, 0.38, and 0.25, respectively. Combinations of gallic acid, ellagic acid, catechin, naringenin, and luteolin with amoxicillin produced additive effects against *S. aureus.* Similarly, gallic acid and ellagic acid in combination with penicillin G also showed additive outcomes. Catechin also showed an additive interaction with cefazolin against *S. aureus.* In contrast, naringenin, and quercetin showed antagonistic interactions in combination with ceftriaxone. Against MRSA, the naringenin-ciprofloxacin combination showed additive interactions, whilst catechin, luteolin or quercetin in combination with cefazolin showed additive interactions.

Since penicillin G showed synergistic interactions against *S. aureus* in combination with catechin, luteolin, and quercetin, this antibiotic was further explored in 50:50 ratios against MRSA. Other β-lactam drugs (amoxicillin, and oxacillin) were also tested. These antibiotics were ineffective against MRSA when evaluated individually. Interestingly, when combined with quercetin at a 50:50 ratio, potential anti-MRSA activity was observed, using a proxy MIC value of 2.5 µg/mL for β-lactams (Table 3). However, synergy was not observed when penicillin G was combined with luteolin or catechin, indicating that the penicillin–quercetin pontentiation is unique in that it synergistically inhibits both *S. aureus* as well its resistant counterpart, MRSA. This contrasts with luteolin and catechin, which, when combined with penicillin G, exert synergistic inhibition of *S. aureus* growth only. This highlights the ability of quercetin to potentiate otherwise inactive antibiotics against resistant strains, and to thereby “reactivate” them to powerfully inhibit both susceptible and resistant *S. aureus.*

### 2.4. Isobologram Analysis

**Figure 1:**
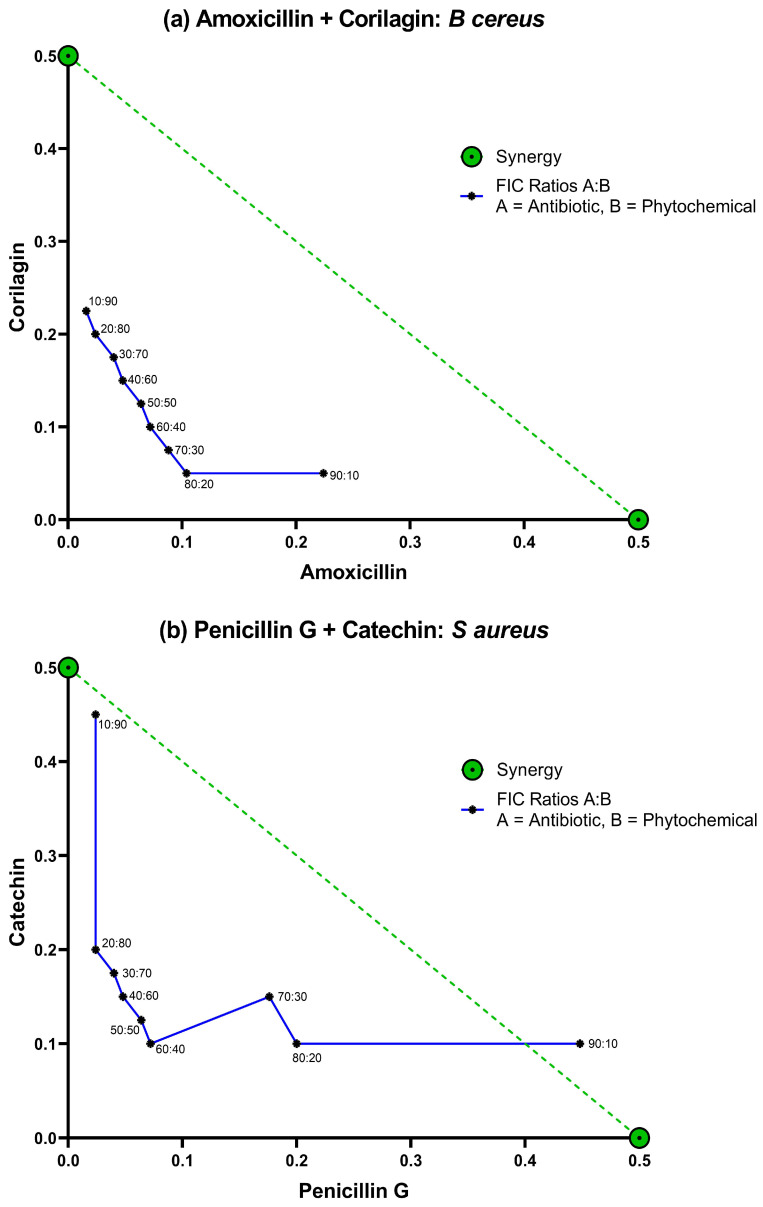

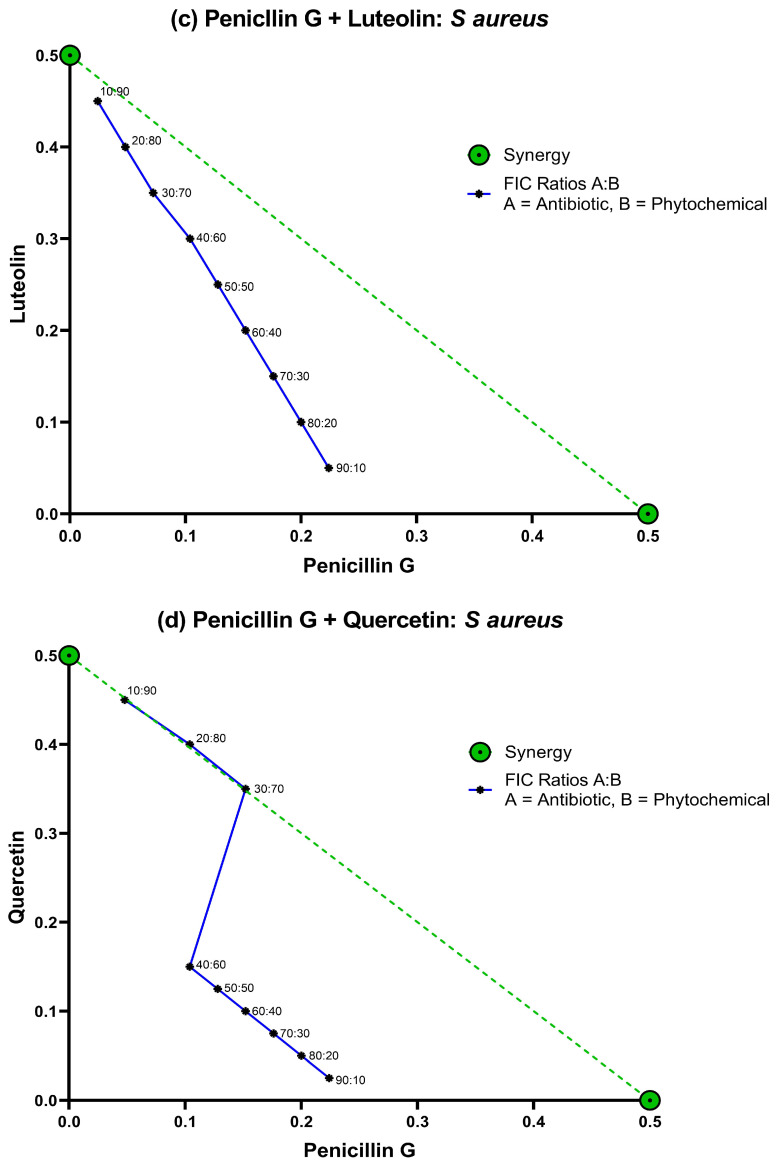

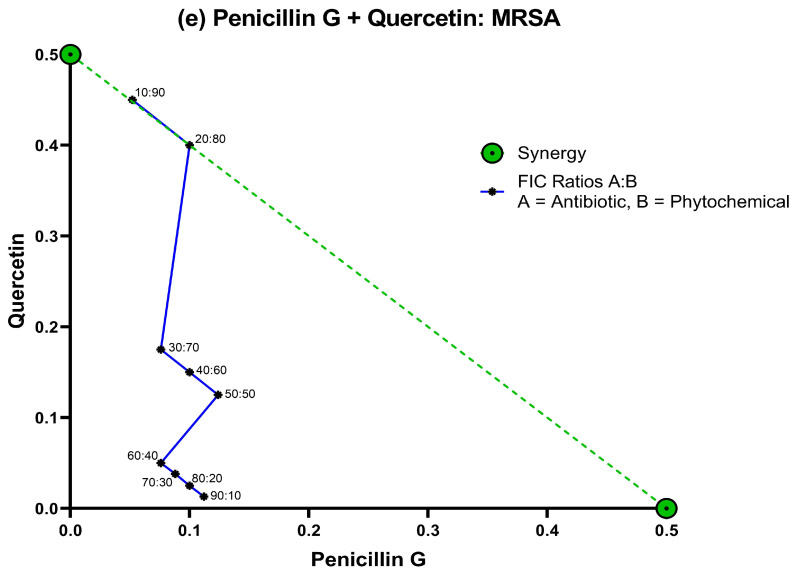
Isobologram analysis of (**a**) amoxicillin–corilagin combinations against *B. cereus*., (**b**) penicillin G–catechin combinations against *S. aureus*., (**c**) penicillin G–luteolin combinations against *S. aureus*., (**d**) penicillin G–quercetin combinations against *S. aureus*., (**e**) penicillin G–quercetin combinations against MRSA. All combinations were at fixed ratios ranging from 10:90 to 90:10 (in 10% increments). The *x*-axis and *y*-axis represent the fractional inhibitory concentrations (FICs) of the antibiotic and phytochemical components respectively. The green broken line of synergy (diagonal) denotes synergy interaction. Data points located below the green line indicate synergy, whereas those above the green line represent additive effects. Each point corresponds to the mean of two independent experiments.

Combination studies revealed that synergy between phytochemicals and antibiotics was ratio dependent. Whilst some antibiotics alone showed no activity against MRSA at 2.5 µg/mL, the use of this concentration as a proxy MIC enabled inclusion in the combination analyses. Certain antibiotic–phytochemical pairs showed strong synergy at specific ratios, effectively restoring or boosting antibiotic efficacy. Table 4 shows the MIC values of selected phytochemicals and antibiotics when tested alone and in combination at different fixed ratios against the bacterial strains *B. cereus*, *S. aureus* and MRSA. Most combinations demonstrated synergistic or additive effects, as reflected by reduced MIC values, significant fold reductions, and FIC index interpretations. Figure 1a–e highlights the graphical representation of different ratios interactions between selected phytochemicals and antibiotics against *B. cereus*, *S. aureus* and MRSA.

Individually, amoxicillin and corilagin showed MICs of 2.5 µg/mL and 62.5 µg/mL, respectively, against *B. cereus*. For *B. cereus* (Figure 1a), combination testing across various ratios (10:90 to 90:10) produced substantial reductions in MICs for both agents, with synergy observed at all ratios. The amoxicillin MIC decreased from 2.5 µg/mL alone to 0.02 µg/mL at 10:90, representing a 63-fold MIC reduction or 63-fold increase in activity. The corilagin MIC decreased from 62.5 µg/mL to 3.1 µg/mL at 80:20 and 90:10, corresponding to a 20-fold MIC reduction or a 20-fold increase in actvity. FIC indices (0.15–0.27) confirmed synergy throughout, demonstrating strong mutual enhancement.

Against S. aureus, robust synergy was also observed with the different penicillin G–phytochemical pairs tested (Table 4). With the catechin (Figure 1b), (MIC = 625 µg/mL), the penicillin G MIC was reduced from 1.25 µg/mL to 0.03 µg/mL at 10:90 and 20:80 (42-fold reduction), whilst the phytochemical MIC declined to 62.5 µg/mL (10-fold reduction) at 80:20 and 90:10 ratios. FIC indices (0.17–0.47) confirmed synergy at most ratios, with only the 90:10 combination yielding additive interaction (FIC = 0.55). In combination with luteolin (Figure 1c) (MIC = 62.5 µg/mL), penicillin G exhibited a 42-fold MIC reduction to 0.03 µg/mL at the 10:90 ratio, whilst the MIC of luteolin decreased 20-fold to 3.1 µg/mL at 90:10. All corresponding FIC indices (0.27–0.47) confirmed synergistic interactions. Similarly, in the presence of quercetin (Figure 1d) (MIC = 1250 µg/mL), the MIC of penicillin G decreased 21-fold to 0.06 µg/mL at 10:90, whereas the MIC of quercetin was reduced 40-fold to 31.3 µg/mL at 90:10. The FIC indices (0.25–0.50) again verified synergy across all tested ratios.

Given the synergistic interaction observed between quercetin and penicillin G against susceptible *S. aureus*, the combination was further evaluated against MRSA. As penicillin G showed no measurable activity within the concentration range tested (up to 2.5 µg/mL), a reference value of 2.5 µg/mL was used to facilitate exploratory interaction analysis. Therefore, any exploratory synergistic interaction observed between quercetin and penicillin G against MRSA should be considered as a poential interaction, which requires further investigation.

The most striking outcome was observed with the MRSA strain, where penicillin G was completely inactive when tested alone. Using a proxy MIC of 2.5 µg/mL for analysis, the antibiotic displayed potential activity in combination with quercetin (Figure 1e), with MICs decreasing to 0.13 µg/mL at 10:90 (19-fold reduction). Quercetin, with an MIC of 2500 µg/mL, was also markedly potentiated, showing an 80-fold MIC reduction or 80-fold increased activity to 31.3 µg/mL at the 90:10 ratio. FIC indices (0.12–0.50) confirmed potentiation across all ratios, demonstrating that the quercetin restored activity to an otherwise inactive antibiotic. Under the experimental conditions tested, the combination produced measurable antibacterial activity, suggesting that the phytochemical may restore or reactivate the antibacterial effect of penillcin G against MRSA; although further studies are required to determine the extent and underlying mechanism of this effect.

### 2.5. β-Lactamase Inhibition Screening of Phytochemicals

**Figure 2:**
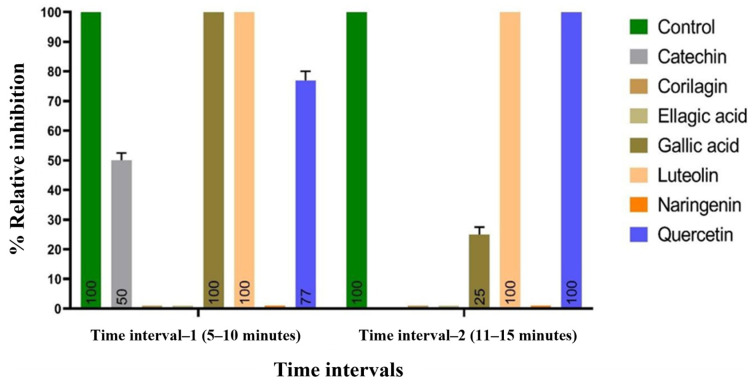
Bar graph represents the percentage relative inhibition (%RI) of β-lactamase activity by phytochemicals (compared to the untreated control) across two-time intervals of 5 min. Data are presented as the mean ± standard deviation (SD) of technical replicates from a single experiment. The numerical %RI values for each phytochemical are shown within the corresponding bars. The β-lactamase assay was performed as a preliminary mechanistic screening assay to compare the relative inhibitory activities of the tested phytochemicals under the experimental conditions employed and should not be interpreted as definitive evidence of the mechanism underlying the observed phytochemical–antibiotic interactions. Note that corilagin, ellagic acid, and naringenin showed no detectable inhibitory activity (0%) during either interval.

The percentage relative inhibition (% RI) of selected phytochemicals on β-lactamase activity were assessed over a linear window of two-time intervals (which were 5 min each): 5–10 min (time interval 1) and 11–15 min (time interval 2). Clavulanic acid was used as a positive control. Figure 2 is intended to provide a comparative overview of the relative β-lactamase inhibitory activities of the tested samples and controls under the assay conditions employed.

Luteolin and quercetin exhibited the strongest and most sustained inhibition of β-lactamase activity (Figure 2). Luteolin maintained 100% inhibition across both time intervals, whilst quercetin showed 85% inhibition at time interval 1 and 100% at time interval 2. Gallic acid displayed complete inhibition (100%) during time interval 1, which decreased to 25% during time interval 2. Catechin produced partial inhibition of 50% at time interval 1 but lost activity completely at time interval 2. Corilagin, ellagic acid, and naringenin showed no detectable inhibitory activity (0%) during either interval.

### 2.6. Toxictiy

**Figure 3:**
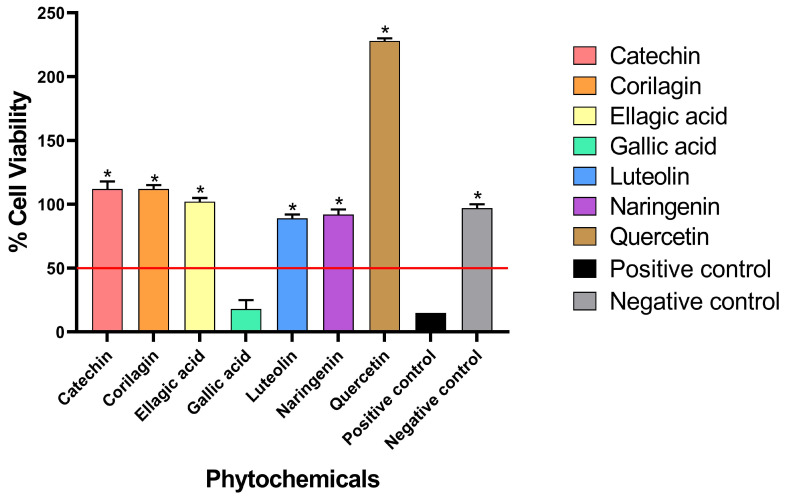
Cell viability percentage of phytochemicals against human dermal adult fibroblasts (HDFs-a). The bar graph shows cell viability (%) following treatment with phytochemicals at either 300 µg/mL (catechin, ellagic acid, naringenin and quercetin) or 75 µg/mL (corilagin, gallic acid and luteolin). The horizontal red line indicates 50% cell viability. Viability ≤ 50% was considered toxic, whereas > 50% indicated non-toxicity. Positive control (docetaxel). Negative control (untreated cells). Data are expressed as mean ± SEM (*n* = 8). Statistical analysis was performed using one-way ANOVA. Differences compared to control were considered statistically significant at *p* < 0.05, represented with *.

The toxicity of phytochemicals was evaluated using human dermal adult fibroblasts (HDFs-a) in cell viability assays. A cell viability of ≤50% was considered toxic, whereas values >50% indicated non-toxicity. Due to differences in compound availability and obtainable stock quantities, pure phytochemicals were evaluated at either 300 µg/mL or 75 µg/mL. These concentrations were selected to enable comparative preliminary screening while conserving limited compounds for subsequent confirmatory assays. Phytochemicals including catechin, ellagic acid, naringenin and quercetin were non-toxic at 300 µg/mL (Figure 3). Notably, quercetin enhanced cell viability to levels exceeding 100% compared to the untreated control. Corilagin, gallic acid, and luteolin were also tested at 75 µg/mL. Noltably, corilagin and luteolin were non-toxic, whereas gallic acid exhibited marked toxicity, comparable to the positive control, docetaxel.

## 3. Discussion

**Figure 4:**
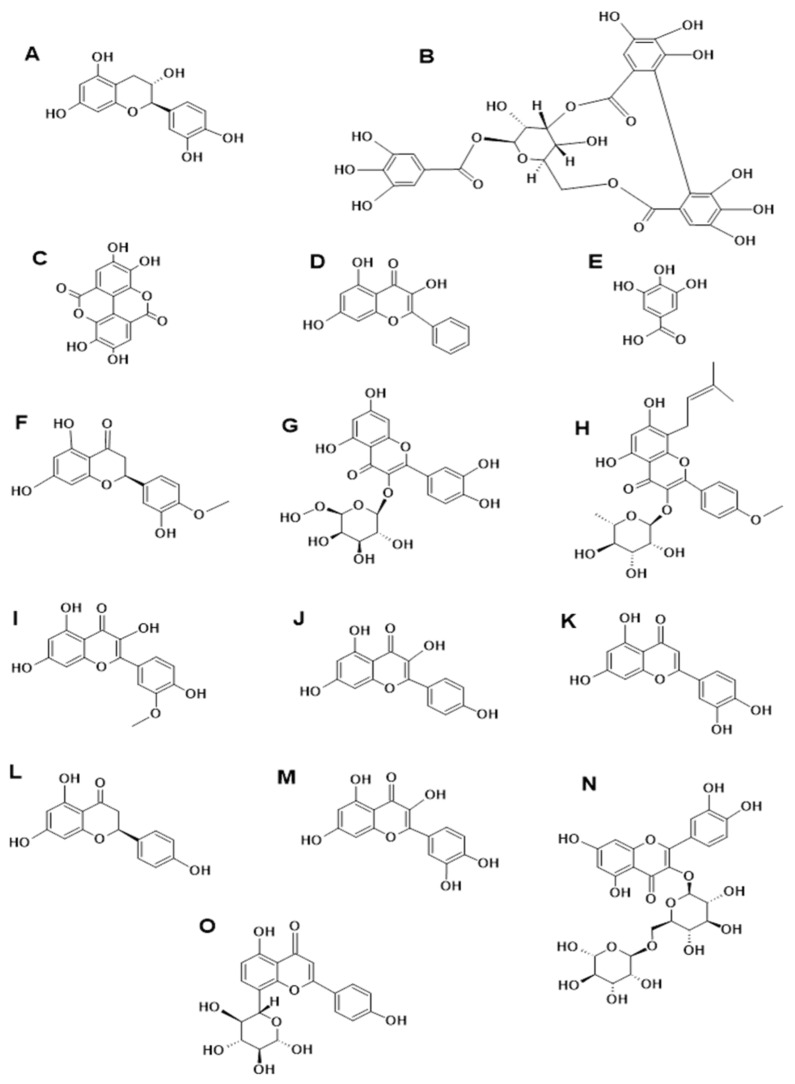
Chemical structures of the phytochemicals evaluated in this study. (**A**) catechin; (**B**) corilagin; (**C**) ellagic acid; (**D**) galangin; (**E**) gallic acid; (**F**) hesperetin; (**G**) hyperoside; (**H**) icariside II; (**I**) isorhamnetin; (**J**) kaempferol; (**K**) luteolin; (**L**) naringenin; (**M**) quercetin; (**N**) rutin; (**O**) vitexin.

This study evaluated the antibacterial potential of selected phytochemicals (Figure 4) against clinically relevant bacterial strains, focusing on their MICs, FIC indices, isobologram analyses, β-lactamase inhibition profiles, and cytotoxicity on HDF-a cell lines. The findings provide insights into both the direct antibacterial effects of the tested compounds and their possible roles as antibiotic adjuvants.

By integrating these results with existing literature, this discussion highlights the therapeutic potential, limitations, and translational implications of these phytochemicals in combating antimicrobial resistance. The antibacterial activities of the tested phytochemicals (Figure 4) revealed distinct differences in potency across bacterial species. Corilagin displayed the strongest inhibition, with MICs of 7.81 µg/mL (12.31 µM) against *S. aureus* and MRSA, and moderate activity against *B. cereus*, *S. flexneri*, and *K. pneumoniae* with MICs of 62.5–250 µg/mL (98–384 µM). These findings agree with earlier reports demonstrating that corilagin markedly potentiates β-lactam activity against MRSA by interfering with PBP2a function and reducing resistance to β-lactams [9,10]. Gallic acid and luteolin also showed moderate activity against *S. aureus* and MRSA (with MICs of 62.5 µg/mL; 368 µM, 218 µM respectively), consistent with values in the literature placing gallic acid MICs at 32–64 µg/mL (188–376 µM) and luteolin MICs within the 16–64 µg/mL (56–224 µM) range depending on strain and medium [11,12,13].

In the present study, naringenin, catechin, ellagic acid and quercetin exhibited comparatively weaker activity, typically within the 625–2500 µg/mL (2295–8273 µM) range, reflecting the limited direct potency of many non-galloylated flavonoids [14,15]. Similar trends have been reported in previous studies, where catechins and related tannins exhibit moderate intrinsic MICs but serve as effective adjuvants by potentiating β-lactams against MRSA [16,17].

Combination analysis provided further insight into the potential of these phytochemicals as antibiotic adjuvants. Amongst the 224 phytochemical–antibiotic pairs, 3.1% were synergistic, 8.5% additive, 83.9% indifferent, and 4.5% antagonistic. Whilst synergy was infrequent, specific combinations yielded clinically meaningful interactions. Against *B. cereus*, corilagin–amoxicillin demonstrated synergy (FIC 0.19), whilst catechin–amoxicillin and naringenin–gentamicin was additive. In *S. sonnei*, several gallic acid combinations with β-lactams and chloramphenicol showed additive outcomes, whereas all combinations against *S. flexneri* were indifferent. Notably, antagonism was observed with catechin–ceftriaxone, gallic acid–gentamicin, and quercetin with several β-lactams against *S. typhimurium*. Therefore, those combinations should be avoided when treating infections of those bacteria. These strain and antibiotic-specific patterns reflect both structural differences among flavonoids and the importance of bacterial resistance mechanisms in shaping pharmacodynamic outcomes [14,18].

Penicillin G combinations with catechin, luteolin, and quercetin demonstrated consistent synergy against *S. aureus*, with FIC indices between 0.19 and 0.38. Similar outcomes have been described previously, where flavonoids were shown to sensitise *S. aureus* to β-lactams by interfering with cell wall synthesis and PBP2a activity [9,13,16]. Quercetin was particularly effective, potentiating penicillin G against MRSA and reducing its MIC from inactivity to 0.13 µg/mL (0.4 µM). These findings are consistent with reports of quercetin and related flavonoids acting as β-lactamase inhibitors and membrane disruptors, thereby restoring β-lactam efficacy in resistant strains [19,20].

Isobologram analyses confirmed that these synergistic interactions were ratio dependent. For *B. cereus*, amoxicillin–corilagin combinations were synergistic across all ratios, with 63-fold and ~20-fold MIC reductions or increased activity for amoxicillin and corilagin, respectively. In *S. aureus*, penicillin G combined with catechin, luteolin, or quercetin yielded 21-42-fold increased antibiotic activity or MIC reductions, alongside reciprocal potentiation of the phytochemicals. The most striking results were obtained in MRSA, where quercetin potentiated penicillin G activity at all ratios tested, with FIC indices between 0.12 and 0.50.

The potentiation observed for penicillin G against MRSA should be interpreted within the context of the experimental design. Because penicillin G exhibited no measurable antibacterial activity within the concentration range evaluated, the assigned reference concentration was used solely for exploratory interpretation of the checkerboard interaction and does not represent a true MIC. Therefore, these findings demonstrate potentiation of penicillin G activity under the experimental conditions tested rather than classical checkerboard synergy. Future studies should determine the complete MIC range of penicillin G against MRSA to further quantify this interaction using conventional FIC methodology. Furthermore, complementary studies, including time–kill assays, are required to determine whether the interaction is bacteriostatic or bactericidal and to further characterise its pharmacodynamic properties.

The β-lactamase inhibition assays provided mechanistic insight into these interactions. Luteolin (100% inhibition at both time intervals) and quercetin (85–100%) displayed strong and sustained inhibition, whilst gallic acid produced rapid but transient inhibition (100% at time interval 1, decreasing to 25% at time interval 2). Catechin showed partial early inhibition (50%) but lost activity at later intervals, whereas corilagin, ellagic acid, and naringenin exhibited no inhibition. These results align with reports demonstrating that quercetin and luteolin inhibit class D β-lactamases including OXA-48 and OXA-98, thereby enhancing β-lactam activity [21,22]. Although the β-lactamase inhibitory activity observed in the present study is consistent with these reports, the commercial β-lactamase assay employed was not designed to identify inhibition of specific β-lactamase classes. Therefore, the present findings should be interpreted as evidence of general β-lactamase inhibitory activity under the experimental conditions employed rather than direct inhibition of class D β-lactamases. In the present study, β-lactamase inhibition assay was included as a preliminary mechanistic screening tool to identify phytochemicals capable of inhibiting β-lactamase activity under the assay conditions employed. Although these findings suggest that β-lactamase inhibition may contribute to the observed antibacterial potentiation of selected phytochemical–antibiotic combinations, additional biochemical and molecular investigations are required to establish a direct mechanistic relationship.

In contrast, the synergistic interactions observed with corilagin and β-lactam antibiotics may involve β-lactamase-independent mechanisms, such as PBP2a inhibition, interference with bacterial cell wall synthesis, and/or efflux pump inhibition [9,10]; however, these potential mechanisms require further investigation.

Phytochemicals that appeared inactive in this study including vitexin, kaempferol, rutin, hesperetin, isorhamnetin, hyperoside, galangin, and icariside II have nevertheless previously been reported to exhibit antibacterial or anti-virulence activity under certain conditions. For example, vitexin displays activity against *S. aureus* with an MIC of approximately 252 µg/mL, although its efficacy varies and is more consistently observed in anti-biofilm assays [18]. Kaempferol typically exhibits MIC values ≥ 512 µg/mL or undetectable activity due to its low solubility, with stronger evidence for antibiofilm effects and aminoglycoside potentiation [16]. Rutin hydrate shows MICs between 128 and 1024 µg/mL against *S. aureus* and MRSA, with additive effects in combination with amikacin [17].

Hyperoside has demonstrated MICs of 128–512 µg/mL against *A. baumannii* [19]. Isorhamnetin, containing honey-derived fractions, has been associated with MIC values of ~256 µg/mL against MDR *S. aureus* (ATCC BAA-44) [20]. Galangin has shown MICs of 50–100 µg/mL against *S. aureus*, with activity linked to membrane damage [23]. Hesperetin derived from hesperidin has antibacterial activity, with MICs 125–500 µg/mL against both Gram-positive and Gram-negative bacteria [24]. However, the study did not clearly describe the preparation or tested concentration of hesperetin and noted poor solubility in 2% DMSO, suggesting that higher DMSO concentrations may be required. In contrast, commercially sourced hesperetin tested in the present study showed no detectable antibacterial activity. These differences may be related to variations in compound solubility, preparation, solvent conditions, or assay methodology. By contrast, icariside II lacks reliable MIC data for antibacterial activity and has primarily been studied previously for other pharmacological effects. Therefore, this is the first study to examine the antibacterial effects of icariside II against susceptible and resistant bacterial pathogens. These findings emphasise that lack of visible activity under one set of conditions does not preclude activity in alternative models, and that assay type, bacterial strain, and structural features such as galloylation heavily influence outcomes [10,11].

Overall, these results support the concept that whilst many phytochemicals have limited direct antibacterial activity on their own, corilagin, luteolin, gallic acid, and quercetin can act as potent adjuvants. Through synergistic interactions, ratio-dependent potentiation, and inhibition of β-lactamase activity, these phytochemicals are capable of re-sensitising resistant strains, restoring the activity of β-lactams, and offering promising leads for combination therapy against multidrug-resistant pathogens.

ESBL *E. coli* was subjected to genome sequencing analysis due to its multidrug-resistant profile, as all tested antibiotics from different classes showed no detectable activity against this strain. Key extended-spectrum β-lactamase genes (*blaCTX-M-64*, *blaTEM-1*, *blaEC-8*) were identified, together with aminoglycoside-modifying enzymes (*aph*, *aadA2*) and the 16S rRNA methyltransferase *rmtB*, which confer high-level aminoglycoside resistance. Resistance determinants for sulfonamides (*sul1*, *sul2*), trimethoprim (*dfrA12*), tetracyclines (*tetA*), macrolides (*mphA*), and phenicols (*floR*) were also present, whilst CARD further revealed an extensive repertoire of efflux pumps (*acrAB-TolC*, *oqxAB*, *mdt*, *emr families*) and global regulators (*marA*, *cpxA*, *evgS/A*, *H-NS*). The phenotypic profile was concordant with these genomic predictions, showing resistance to nearly all clinically relevant antibiotics tested, with only polymyxin B retaining very low activity at MIC of 2.5 µg/mL. This highlights the substantial therapeutic challenge posed by this strain and underscores the urgency of exploring novel treatment strategies.

From the tested phytochemicals, only gallic acid also exhibited antibacterial activity against ESBL *K. pneumoniae* and ESBL *E. coli*, with MICs of 625 and 1250 µg/mL, respectively. The observed inhibitory activity can be linked to the known mechanisms of phytochemicals. Phenolic compounds such as gallic acid are reported to disrupt bacterial membranes, generate oxidative stress, and inhibit critical enzymes, potentially bypassing conventional resistance determinants [1,2,3,14]. Collectively, these findings highlight the therapeutic potential of the selected phytochemicals against tested bacterial pathogens. Although their MIC values were generally higher than those of conventional antibiotics, the observed antibacterial activity and phytochemical–antibiotic interactions against these strains are encouraging. The mechanisms proposed in the present study, including β-lactamase inhibition, PBP2a inhibition, and efflux pump inhibition, are based on the observed biological activities together with evidence from previous studies and should therefore be regarded as plausible hypotheses rather than definitive mechanistic conclusions. Future studies should investigate these mechanisms using complementary approaches, including molecular docking, target-binding assays, enzyme kinetic analyses, and efflux pump inhibition studies, to further elucidate the molecular basis of the observed antibacterial potentiation and support the translation of these phytochemicals as potential antibiotic adjuvants for combating antimicrobial resistance.

Although DMSO is widely used as a solvent in in vitro assays, it was employed at low concentrations and tested in parallel with appropriate vehicle controls in this study. However, DMSO can influence cell membrane structure and permeability. Such effects may potentially alter intracellular access of test compounds compared to a DMSO-free system. This represents a general limitation of in vitro assays employing DMSO as a solvent and has been discussed previously in the literature [25]. In parallel, phytochemicals catechin, ellagic acid, and naringenin were also non-toxic at 300 µg/mL. Notably, quercetin elevated cell viability to >100%, indicating a potential cytoprotective or proliferative effect. Viability values exceeding 100% may reflect increased cellular metabolic activity relative to untreated controls. However, because the resazurin assay measures cellular metabolic activity rather than direct cell number or proliferation, these findings should be regarded as preliminary observations and should not be interpreted as definitive evidence of a cytoprotective or proliferative effect without confirmation using complementary assays.

The observation that quercetin enhances cell viability aligns with prior studies showing that quercetin can exert cytoprotective effects via activation of mitochondrial potassium channels and antioxidant pathways, thereby improving endothelial or fibroblast survival under stress conditions [26]. In human lung fibroblasts, quercetin was found to extend cellular viability and influence membrane homeostasis, supporting its role in promoting cell survival [27]. Furthermore, quercetin has been identified as a proteasome activator with antioxidant properties, which can enhance cellular lifespan and resilience in normal fibroblasts [27].

Because of solubility or potency constraints, some phytochemicals (corilagin, gallic acid, luteolin) were tested at a lower concentration of 75 µg/mL. At this lower dose, corilagin and luteolin were non-toxic. However, gallic acid demonstrated cytotoxicity comparable to the positive control, docetaxel. Docetaxel is a well-known chemotherapeutic agent associated with cytotoxic effects in proliferative and non-target tissues [28,29]. Thus, gallic acid’s similarity in toxicity to docetaxel highlights the need for careful dose optimisation, structural refinement, or targeted delivery before therapeutic use.

Overall, selected phytochemicals appear to maintain non-toxic profile in dermal fibroblast assays. The dual profile of quercetin as non-toxic and viability-enhancing positions it as a particularly promising candidate for formulations aiming at both bioactivity and cellular support (e.g., wound healing, skin regeneration). The observed toxicity of gallic acid at 75 µg/mL highlights the importance of evaluating the safety profiles of phytochemicals and supports the need for further dose-dependent toxicity assessment in future studies.

Future studies should be geared towards addressing the limitations of our study in which a proxy MIC value was required in order to calculate some of the FIC values for quercetin’s effects on MRSA. This may be resolved using time–kill bacterial inhibition studies or the use of higher concentrations of antibiotics, although we were careful in this study to avoid using antibiotic concentrations that would be equivalent to supra-pharmacological doses in vivo. Additionally, future work could also involve assessing the inhibitory effects of quercetin and other phytochemicals against different classes of β-lactamase enzymes. Findings from such work may then lead to molecular docking and other ligand–enzyme structural studies.

## 4. Materials and Methods

### 4.1. Phytochemicals

All phytochemicals (Table 5) were reconstituted in 10% dimethyl sulfoxide (DMSO) to prepare stock solutions of 1 mg/mL (gallic acid, corilagin, luteolin, vitexin, kaempferol, hesperetin, rutin hydrate, isorhamnetin, hyperoside, galangin and icariside II) and 10 mg/mL (gallic acid, ellagic acid, catechin hydrate, naringenin, and quercetin) in 10 mL. All compounds were of analytical or reference grade and purchased from Merck Life Science Pty Ltd. (Bayswater, Australia).

### 4.2. Antibiotics and Bacerial Srains

Powdered antibiotics used in this study included penicillin G (potency 1440–1680 µg/mg), ciprofloxacin (≥98% purity by HPLC), polymyxin B (purity > 90%), oxacillin (≥95% purity by TLC), dicloxacillin (≥98% purity by HPLC), amoxicillin (potency of 900 µg/mg), ticarcillin (potency of 800 µg/mg), cefazolin (≥96% purity by HPLC), ceftriaxone (≥97% purity by HPLC), erythromycin (potency ≥ 850 µg/mg), tetracycline (≥95% purity by HPLC), chloramphenicol (≥98% purity by HPLC), gentamicin (≥98% purity by HPLC) and vancomycin (≥95% purity by TLC) (Merck Life Science Pty. Ltd., Melbourne, Australia). Stock solutions of powdered antibiotics were prepared in autoclaved ultrapure water at 1 mg/mL for microdilution assays and stored at −20 °C. Chloramphenicol, tetracycline, and erythromycin were dissolved in 70% ethanol, whilst amoxicillin was prepared in 0.1 M sodium phosphate buffer. The antibiotics were selected based on their clinical relevance to the bacterial pathogens investigated and their representation of different antimicrobial classes and mechanisms. Additionally, they were chosen for their suitability in evaluating phytochemical–antibiotic interactions and potential resistance-modulating effects.

Reference bacterial strains included *Shigella sonnei* (ATCC 25931), *Salmonella enterica* serovar *Typhimurium* (ATCC 14028), *Shigella flexneri* (ATCC 12022), *Bacillus cereus* (ATCC 14579), *Klebsiella pneumoniae* (ATCC 13883), extended-spectrum β-lactamase (ESBL) *K. pneumoniae* (ATCC 700603), *Escherichia coli* (ATCC 25922), *Staphylococcus aureus* (ATCC 25923), and methicillin-resistant *S. aureus* (MRSA) (ATCC 43300) were sourced from the American Type Culture Collection (ATCC) through In Vitro Technologies (Melbourne, Australia). Additionally, an ESBL-producing *E. coli* clinical isolate was sourced from the Gold Coast University Hospital (Southport, Australia). Cultures were maintained on Mueller–Hinton (MH) agar and broth (Oxoid Ltd., Adelaide, Australia), with MRSA grown at 35 °C and all other strains at 37 °C for 18–24 h.

Whole-genome sequencing data of the ESBL *E. coli* isolate were analysed using three established antimicrobial resistance databases. These were the Comprehensive Antibiotic Resistance Database (CARD), NCBI AMR Finder, and ResFinder. The analyses were conducted by Queensland Public Health and Infectious diseases Reference Genomics (Q-PHIRE Genomics; Queensland Health, Brisbane, Australia). Raw sequence reads were processed and annotated against each database to identify resistance determinants, with cross-verified results to ensure accuracy. Genes were categorised according to their resistance mechanisms, including β-lactamases, aminoglycoside-modifying enzymes, efflux pumps, and regulatory elements. The combined outputs provided a comprehensive genetic resistance profile that was compared with the phenotypic antibiotic susceptibility testing results.

### 4.3. Calculation of Minimum Inhibitory Concentration

Minimum inhibitory concentrations (MICs) of the phytochemicals and reference antibiotics were assessed through a 96-well plate microdilution assay [30]. In each well, 100 μL of MH broth was dispensed, and the first well of each row received 100 μL of phytochemical or antibiotic solution. Serial two-fold dilutions were then prepared across the row. Each well was subsequently inoculated with 100 μL of a 1:100 dilution of a 0.5 McFarland bacterial suspension, except for the sterile controls to which no bacteria was added. Plates were incubated at 37 °C (MRSA at 35 °C) for 20–24 h. To calculate the MICs of phytochemicals, the ρ-iodonitrotetrazolium violet (INT; Sigma Aldrich, Melbourne, Australia) assay was employed. A 40 µL aliquot of INT (final concentration: 0.4 mg/mL) was added to parallel wells, where metabolically active bacteria reduced the colourless INT to an insoluble red formazan, confirming bacterial growth. The MIC was determined as the lowest concentration of the phytochemicals that completely inhibited bacterial growth, indicated by the absence of a red–pink coloration. MIC determinations were performed in duplicate wells within a single experiment, and the reported values represent the results obtained from these technical replicates.

Phytochemical stock solutions were prepared in 10% (*v*/*v*) DMSO and subsequently diluted in Mueller–Hinton broth for MIC determination. The highest final DMSO concentration present in the assay was 2.5% in the first test well, decreasing further with serial two-fold dilutions. Wells containing equivalent concentrations of DMSO without phytochemicals were included as vehicle controls to assess any potential effects of the solvent on bacterial growth.

### 4.4. Fractional Inhibitory Concentration Analysis

Phytochemicals and antibiotics showing antibacterial effects (MIC ≤ 2500 μg/mL for phytochemicals; ≤2.5 μg/mL for antibiotics) were selected for subsequent combination testing. Phytochemical–antibiotics in equal volumes (1:1 ratio) were added into wells, and their effects against the bacterial panel were assessed using the fractional inhibitory concentration (ΣFIC) approach. The ΣFIC values for each combination were calculated using the following formulas:

FIC(A) = (MIC of A in combination)/(MIC of A alone)

FIC(B)=(MIC of B in combination)/(MIC of B alone)

ΣFIC = FIC(A) + FIC(B)

Outcomes were interpreted as follows: synergistic when ΣFIC ≤ 0.5, additive when 0.5 < ΣFIC ≤ 1.0, indifferent when 1.0 < ΣFIC ≤ 4.0, and antagonistic when ΣFIC > 4.0 [31]. Additionally, for antibiotics that did not exhibit measurable activity at the highest tested concentration (2.5 µg/mL), a proxy MIC of 2.5 µg/mL was assigned to enable FIC index calculation. This allowed assessment of potential synergistic or reactivating effects when these antibiotics were tested in combination with phytochemicals against MRSA.

### 4.5. Isobologram Analysis

To investigate combinations that exhibit synergy, their optimal ratios were determined. The same procedure as described in the liquid dilution assay was followed [30], with variations in the ratios of phytochemical–antibiotic combinations tested. The ratio of conventional antibiotics to phytochemicals was sequentially reduced by 10 μL from an initial volume of 90 μL, ultimately reaching 10 μL, creating combinations ranging from 90% to 10% antibiotic with 10% decreasing increments. Simultaneously, the amount of phytochemical was increased by 10 μL, starting from 10 μL and reaching 90 μL. For penicillin G that showed no measurable activity at 2.5 µg/mL against MRSA, a proxy MIC >2.5 µg/mL was assigned to enable inclusion in isobologram construction and FIC index calculations. This approach allowed consistent assessment of synergistic or reactivating interactions at different combination ratios.

All assays were conducted in duplicates. The acquired data were used to calculate FIC values, and an isobologram analysis was employed to determine the ratios at which a synergistic interaction occurred between phytochemicals and antibiotics. Interactions resulting in a ΣFIC value of ≤0.5 were considered synergistic, whilst ΣFIC values between 0.5 and 1.0 were considered additive. Interactions with ΣFIC values greater than 1.0 but not exceeding 4.0 were classified as non-interactive, and interactions with ΣFIC values greater than 4.0 were deemed antagonistic.

### 4.6. β-Lactamase Inhibition Screening

The seven phytochemicals were tested for β-lactamase inhibition activity using a commercial β-lactamase assay kit (βL; EC 3.5.2.6) (Sigma-Aldrich, Melbourne, Australia) based on a previously described protocol [32]. Each sample was prepared at a total volume of 200 µL for duplicate testing. The final concentration in the assay wells was 50 µg/mL for phytochemicals. DMSO percentages were calculated to ensure they remained 0.5% in the wells. To achieve the desired final concentrations, 5X concentrated working solutions of each phytochemical was prepared. For phytochemical compounds with a target final concentration of 50 µg/mL, 5X concentrated working solutions were prepared at 250 µg/mL. A uniform concentration of 50 µg/mL was selected for the β-lactamase inhibition assay to enable direct comparison of the relative inhibitory activities of all phytochemicals under identical screening conditions, irrespective of their individual antibacterial MIC values.

Phytochemicals, including quercetin, ellagic acid, gallic acid, catechin, and naringenin, were prepared at a stock concentration of 500 µg/mL, and were derived from primary stock solutions of 10 mg/mL prepared in 10% DMSO. Corilagin and luteolin were prepared at a stock concentration of 250 µg/mL and were prepared from primary stock solutions of 1 mg/mL prepared in 10% DMSO.

A volume of 20 µL of test components were dispensed into duplicate wells of a 96-well plate. In the assay, the “sample inhibitor” refers to the sample (pure phytochemical) being tested. The experimental setup included: (i) sample inhibitor (5X inhibitor solution), (ii) sample blank (5X inhibitor solution without enzyme), (iii) enzyme control (β-lactamase assay buffer), and (iv) control wells containing either the reference inhibitor (clavulanic acid) or vehicle (DMSO).

For the inhibition reaction, enzyme was diluted 1 in 25 in the assay buffer. The mixture was added to wells containing sample inhibitors, enzyme controls, inhibitor controls, or vehicle controls. In the case of sample blanks or vehicle blanks, 50 µL of β-lactamase assay buffer alone was used. Each well therefore received 50 µL of the appropriate inhibition reaction mixture. The plate was mixed thoroughly and then incubated at 25 °C for 10 min, protected from light by covering it with aluminium foil. The enzymatic reaction mixture was prepared by combining 29 µL of β-lactamase assay buffer with 1 µL of nitrocefin, yielding 30 µL per well. This mixture was added to each well and mixed thoroughly.

The enzymatic activity was monitored by measuring absorbances at 490 nm using a microplate reader in kinetic mode for 30 min, with measurements recorded each minute. Data collection and analysis were performed using the Tecan i-control 2.0 software on an Infinite 200 Pro system. Two-time intervals [time intervals of 5–10 min (T1), followed by time intervals of 11–15 min (T2)] were selected from linear window of 5 min to 15 min for the calculation of % relative inhibition.

The sample inhibitor tested the inhibitory effect of each sample on β-lactamase activity. The sample blank contained the same sample without the enzyme and was used to account for any background absorbance from sample readings. The enzyme control contained only β-lactamase and buffer (without inhibitor) and represented 100% enzyme activity, serving as the baseline for inhibition calculations. The inhibitor control included a β-lactamase inhibitor (clavulanic acid) to confirm that the assay effectively detected enzyme inhibition. The vehicle control contained DMSO, used as the solvent, with its maximum concentration limited to 0.8% for phytochemicals.

To calculate the percentage of relative inhibition (% RI), the absorbance (ABS) for each well was plotted against time. Two time points (T_1_ and T_2_) within the linear range (T_1_ = 5–10 min; T2 = 11–15 min) of the curve were selected to determine the slope for each well between T_1_ and T_2_. The corresponding absorbance values (ABS_1_ and ABS_2_) were used to calculate the reaction rate (ABS/minute). An enzyme control was included in each assay run to ensure consistency. The slope of the sample blank was subtracted from that of the corresponding sample inhibitor at each time point to obtain the slope of corrected measurement (Slope CM), which was then used to calculate the % relative inhibition.

The slope of the corrected measurement (Slope CM) represents the rate obtained from the sample inhibitor, while the slope of the enzyme control (Slope EC) corresponds to the β-lactamase activity. Irreversible inhibitors, such as clavulanic acid, completely suppress β-lactamase activity, resulting in ΔABS = 0 and a % relative inhibition of 100%. Negative values were adjusted to 0%, and results exceeding 100% were set to 100%. It should be noted that these values are relative to the measured absorbance and do not represent absolute inhibition. The data shown represents technical replicates within a single experiment run, with error bars indicating standard deviation (SD) of experimental variability. Technical replicates refer to repeated measurements performed under identical experimental conditions within a single experiment to assess assay precision.

Slope = (ABS2 − ABS1)/(T2 − T1) = ΔABS/minute

Slope CM = (Slope Phytochemical + Enzyme) − (Slope Phytochemical only)

%RI = Slope EC − Slope CM/Slope EC × 100

### 4.7. Toxicity in Adult Human Dermal Fibroblasts

Adult human dermal fibroblasts (HDFs-a) were used for toxicity analyses and followed previously published methodologies [33,34]. Cells at passage 5 were used, following routine subculture, to confirm healthy morphology. Cell lines were grown in complete medium (fibroblast growth medium; catalogue no. C0135C, supplemented with low serum growth supplement; catalogue no. S00310) purchased from Thermo Fisher Scientific Australia Pty. Ltd. Cells were expanded and counted using a Countess 3 Automated Cell Counter (Thermo Fisher Scientific, Brisbane, Australia) and the suspension was adjusted to 50,000 cells/mL. A 100 µL of the cell suspension (5000 cells/well) was dispensed into each well of a 96-well flat-bottom pate, followed by 40 µL of complete medium to reach 140 µL/well. Plates were incubated for 24 h at 37 °C in an atmosphere of 5% CO_2_.

For the pure compounds, quercetin, ellagic acid, catechin and naringenin were tested at 300 µg/mL, prepared from 1 mg/mL stocks in 1% DMSO. The other three phytochemicals (corilagin, gallic acid, luteolin) were tested at 75 µg/mL from 0.25 mg/mL stocks in 2.5% DMSO. All phytochemicals and DMSO dilutions were prepared in autoclaved ultrapure water. Due to the limited availability of several purified phytochemicals, cytotoxicity assays were performed at either 300 or 75 µg/mL, depending on compound availability. These concentrations were selected to provide an initial in vitro cytocompatibility assessment rather than for direct quantitative comparison between all tested phytochemicals. For dosing, 60 µL of each phytochemical solution was added to the appropriate wells, while for the positive control, 20 µL of 1 mM docetaxel plus 40 µL of complete medium were added. Plates were incubated for a further 24 h.

Following aspiration of culture medium (200 µL/well), 200 µL of a 44 mM resazurin solution was added to each well. Plates were incubated for 1 h and absorbances immediately recorded using a Tecan Infinite 200 Pro fluorescence plate reader equipped with i-control 2.0 software for fluorescence and absorbance measurements. Recordings were taken every hour for 6 h. Average signals were quantified relative to the appropriate controls (DMSO, media), converted to percent response as cell viability after correction with the appropriate controls, and summarised graphically. All conditions were run in technical quadruplicate, and the experiment repeated on two separate days (*n* = 8).

The % cell viability could then be calculated using the following formula:

$$\%\;Cell\;Viability\;for\;phytochemicals=\frac{Phytochemical\;exposure}{Media\;control}\times 100$$

$$\%\;Cell\;Viability\;for\;DMSO=\frac{DMSO\;vehicle\;control\;exposure}{Media\;control}\times 100$$

To evaluate DMSO cytotoxicity, the cell viability of DMSO control was first expressed relative to the media control, and for samples (phytochemicals) the corrected viability (%) was obtained by normalising the sample viability to the DMSO control viability, using the following formula:

$$\%\;Corrected\;Cell\;Viability\;of\;samples=\frac{Viability\;\%\;(Samples)\;}{Viability\;\%\;(DMSO)}\;\times \;100$$

This approach eliminates the contribution of DMSO toxicity and reflects compound-specific effects on cell viability. A cell viability threshold of ≤50% was used to indicate cytotoxicity, consistent with common in vitro screening practices. This classification reflects concentration-dependent effects observed under experimental conditions and is intended for comparative assessment rather than direct extrapolation to in vitro toxicity.

Corrected cell viability percentage of phytochemicals are presented as bar graphs showing mean ± SEM, calculated from quadruplicate technical replicates performed across two independent experiments conducted on separate days (*n* = 8). Statistical comparisons between multiple treatment groups and control were performed using one-way analysis of variance (ANOVA), with *p* < 0.05 considered statistically significant. Phytochemicals with cell viability ≤ 50% were considered toxic, whereas those with cell viability > 50% were deemed non-toxic.

## 5. Conclusions

This study demonstrates that selected phytochemicals possess significant potential as antibacterial agents and more importantly, as resistance-modifying adjuvants. Whilst the intrinsic antibacterial activity varied, corilagin, luteolin, gallic acid, and quercetin exhibited notable MIC values against *S. aureus* and MRSA. Combination testing revealed that although most phytochemical–antibiotic interactions were indifferent, key pairings produced robust synergy, particularly between flavonoids and β-lactams, resulting in substantial fold increased in antibiotics activity. Isobologram analyses further emphasised the ratio dependency of these interactions, with quercetin notably restoring penicillin G activity against MRSA at otherwise inactive concentrations.

Mechanistic assays indicated that luteolin and quercetin provided strong and sustained β-lactamase inhibition, whilst gallic acid and catechin showed transient effects demonstrated potent time-dependent inhibition. These findings support the dual role of phytochemicals direct antibacterial agents and enzymatic inhibitors, capable of enhancing the efficacy of β-lactams against resistant strains.

Overall, the findings demonstrate that some selected phytochemicals possess antibacterial activity and, as evidenced by the FIC and isobologram analyses, several phytochemical–antibiotic combinations exhibited synergistic or additive interactions that enhanced the activity of conventional antibiotics against susceptible and resistant bacterial pathogens. These findings highlight the potential of phytochemical-based strategies as antibiotic adjuvants to improve antibacterial efficacy and may contribute to overcoming antimicrobial resistance through complementary mechanisms. Further investigation of the underlying mechanisms of action, structure–activity relationships, pharmacokinetic properties, and safety profiles is warranted to support the future development and clinical translation of these promising compounds.

## Figures and Tables

**Table 1:** Antibacterial activity as minimum inhibitory concentration (MIC) of phytochemicals and antibiotics against panel of bacterial pathogens.

Phytochemicals or Antibiotics	Bacterial Strains & MIC in µg/mL (and in µM)									
	*S. aureus*	MRSA	*E. coli*	ESBL *E. coli*	*K. pneumoniae*	ESBL *K. pneumoniae*	*B. cereus*	*S. flexneri*	*S. sonnei*	*S. typhimurium*
GA	**62.5** **(** **367.56** **)**	**62.5** **(** **367.56** **)**	**1250** **(** **7348.6** **)**	**1250** **(** **7348.6** **)**	**312.5** **(** **1837.15** **)**	**625** **(** **3674.3** **)**	**625** **(** **3674.3** **)**	**625** **(** **3674.3** **)**	**1250** **(** **7348.6** **)**	**625** **(** **3674.3** **)**
EA	**625** **(** **2068.41** **)**	-	-	-	-	-	-	**625** **(** **2068.41** **)**	**1250** **(** **4136.82** **)**	**625** **(** **2068.41** **)**
CAT	**625** **(** **2026.5** **)**	**625** **(** **2026.5** **)**	-	-	-	-	**1250** **(** **4053** **)**	**1250** **(** **4053** **)**	**1250** **(** **4053** **)**	**1250** **(** **4053** **)**
NAR	**625** **(** **2295.07** **)**	**625** **(** **2295.07** **)**	-	-	-	-	**625** **(** **2295.07** **)**	-	-	-
VIT	-	-	-	-	-	-	-	-	-	-
KAM	-	-	-	-	-	-	-	-	-	-
LUT	**62.5** **(** **218.38** **)**	**62.5** **(** **218.38** **)**	-	-	-	-	-	-	-	-
COR	**7.81** **(** **12.31** **)**	**7.81** **(** **12.31** **)**	-	-	**250** **(** **393.86** **)**	-	**62.5** **(** **98.47** **)**	**125** **(** **196.93** **)**	-	-
HES	-	-	-	-	-	-	-	-	-	-
RUT	-	-	-	-	-	-	-	-	-	-
ISO	-	-	-	-	-	-	-	-	-	-
QUE	**1250** **(** **2068.23** **)**	**2500** **(** **8272.92** **)**	-	-	-	-	-	**625** **(** **2068.23** **)**	**1250** **(** **4136.46** **)**	**625** **(** **2068.23** **)**
HYP	-	-	-	-	-	-	-	-	-	-
GIN	-	-	-	-	-	-	-	-	-	-
ICA	-	-	-	-	-	-	-	-	-	-
**ANTIBIOTICS**										
PB	-	-	**2.5** **(** **2.08** **)**	**2.5** **(** **2.08** **)**	**1.25** **(** **1.04** **)**	-	-	**0.02** **(** **0.08** **)**	**0.02** **(** **0.08** **)**	**0.02** **(** **0.08** **)**
CIP	**0.63** **(** **1.90** **)**	**0.63** **(** **1.90** **)**	**0.02** **(** **0.06** **)**	-	**0.02** **(** **0.06** **)**	**0.63** **(** **1.90** **)**	**0.16** **(** **0.48** **)**	**0.02** **(** **0.06** **)**	**0.02** **(** **0.06** **)**	**0.02** **(** **0.06** **)**
VAN	**1.25** **(** **0.86** **)**	**0.63** **(** **0.43** **)**	-	-	-	-	**0.63** **(** **0.43** **)**	-	-	-
GEN	-	-	**1.25** **(** **2.62** **)**	-	**0.31** **(** **0.65** **)**	-	**0.31** **(** **0.65** **)**	**1.25** **(** **2.62** **)**	**2.5** **(** **5.23** **)**	**2.5** **(** **5.23** **)**
AMX	**1.25** **(** **3.42** **)**	-	**2.5** **(** **6.84** **)**	-	-	-	**2.5** **(** **6.84** **)**	**2.5** **(** **6.84** **)**	**2.5** **(** **6.84** **)**	**2.5** **(** **6.84** **)**
CZ	**0.63** **(** **1.39** **)**	-	**2.5** **(** **5.50** **)**	-	**2.5** **(** **5.50** **)**	-	-	**2.5** **(** **5.50** **)**	**1.25** **(** **2.75** **)**	**1.25** **(** **2.75** **)**
TIC	-	-	**2.5** **(** **6.50** **)**	-	-	-	-	**2.5** **(** **6.50** **)**	-	-
CT	**2.5** **(** **4.51** **)**	-	**0.31** **(** **0.56** **)**	-	**0.31** **(** **0.56** **)**	-	-	**0.16** **(** **0.29** **)**	**0.16** **(** **0.29** **)**	**0.16** **(** **0.29** **)**
OX	**0.16** **(** **0.40** **)**	-	-	-	-	-	-	-	-	-
DI	**0.16** **(** **0.34** **)**	**2.5** **(** **5.32** **)**	**2.5** **(** **5.32** **)**	**2.5** **(** **5.32** **)**	-	-	-	-	-	-
PG	**1.25** **(** **3.74** **)**	-	-	-	-	-	-	-	-	-
ERY	**0.31** **(** **0.42** **)**	-	-	-	-	-	**0.16** **(** **0.22** **)**	-	-	-
TET	**0.08** **(** **0.18** **)**	**0.04** **(** **0.09** **)**	**0.31** **(** **0.70** **)**	-	**0.31** **(** **0.70** **)**	-	**0.02** **(** **0.05** **)**	**0.31** **(** **0.70** **)**	**0.31** **(** **0.70** **)**	**0.31** **(** **0.70** **)**
CHL	-	-	**2.5** **(** **7.74** **)**	-	**2.5** **(** **7.74** **)**	-	-	**1.25** **(** **3.87** **)**	**2.5** **(** **7.74** **)**	-

GA = gallic acid, EA = ellagic acid, CAT = (+)-catechin hydrate, NAR = (+/−)-naringenin, VIT = vitexin, KAM = kaempferol, LUT = luteolin, COR = corilagin, HES = hesperetin, RUT = rutin hydrate, ISO = isorhamnetin, QUE = quercetin, HYP = hyperoside, GIN = galangin, ICA = icariside II, PB = polymyxin B, CIP = ciprofloxacin, VAN = vancomycin, GEN = gentamicin, AMX = amoxycillin, CZ = cefazolin, TIC = ticarcillin, CT = ceftriaxone, OXA = oxacillin, DI = dicloxacillin, PG = penicillin G, ERY = erythromycin, TET = tetracycline, and CHL = chloramphenicol. Active phytochemicals and antibiotics are shown in **bold**, whilst a lack of antibiotic activity was observed when MIC values > 2.5 µg/mL. Phytochemicals and antibiotics with no antibacterial activity were represented as a dash (-).The red numbers are MIC values expressed in µM.

**Table 2:** Antimicrobial resistance analysis of the ESBL *Escherichia coli* species using NCBI and ResFinder gene identifications following whole genome sequencing.

NCBI Analysis					
Gene	Coverage	%Identity	Accession	Product	Resistance
blaCTX-M-64	1–876/876	100	NG_049015.1	class A extended-spectrum beta-lactamase CTX-M-64	Cephalosporin
aph(3′)-IIa	1–795/795	100	NG_047417.1	aminoglycoside O-phosphotransferase APH(3′)- IIa	Kanamycin
blaTEM-1	1–861/861	100	NG_050145.1	class A broad-spectrum beta-lactamase TEM-1	Beta-lactam
rmtB1	1–756/756	100	NG_048058.1	16S rRNA (guanine(1405)-N(7))- methyltransferase RmtB1	Aminoglycoside
sul1	1–840/840	100	NG_048082.1	sulfonamide-resistant dihydropteroate synthase Sul1	Sulfonamide
aadA2	1–792/792	100	NG_047343.1	ANT(3″)-Ia family aminoglycoside nucleotidyltransferase AadA2	Streptomycin
dfrA12	1–498/498	100	NG_047689.1	trimethoprim-resistant dihydrofolate reductase DfrA12	Trimethoprim
floR	1–1215/1215	99.83	NG_047869.1	chloramphenicol/florfenicol efflux MFS transporter FloR	Chloramphenicol; florfenicol
tet(A)	1–1200/1200	99.92	NG_048153.1	tetracycline efflux MFS transporter Tet(A)	Tetracycline
aph(6)-Id	1–837/837	100	NG_047466.1	aminoglycoside O-phosphotransferase APH(6)-Id	Streptomycin
aph(3**″**)-Ib	1–828/828	100	NG_056002.2	aminoglycoside O-phosphotransferase APH(3″)-Ib	Streptomycin
sul2	1–816/816	100	NG_051852.1	sulfonamide-resistant dihydropteroate synthase Sul2	Sulfonamide
oqxA	1–1176/1176	100	NG_048024.1	multidrug efflux RND transporter periplasmic adaptor subunit OqxA	Phenicol; quinolone
oqxB	1–3153/3153	100	NG_048025.1	multidrug efflux RND transporter permease subunit OqxB	Phenicol; quinolone
mph(A)	1–921/921	99.67	NG_047986.1	Mph(A) family macrolide 2′-phosphotransferase	Macrolide
blaEC-8	1–1134/1134	98.33	NG_049086.1	cephalosporin-hydrolyzing class C beta-lactamase EC-8	Cephalosporin
Resfinder analysis					
blaCTX-M-64_1	1–876/876	100	AB284167	blaCTX-M-64	Amoxicillin; Ampicillin; Aztreonam; Cefepime; Cefotaxime; Ceftazidime; Ceftriaxone; Piperacillin; Ticarcillin
aph(3′)-IIa_2	1–795/795	100	V00618	aph(3′)-IIa	Kanamycin; Neomycin
blaTEM-1B_1	1–861/861	100	AY458016	blaTEM-1B	Amoxicillin; Ampicillin; Cephalothin; Piperacillin; Ticarcillin
rmtB_1	1–756/756	100	AB103506	rmtB	Amikacin; Arbekacin; Gentamicin; Isepamicin; Kanamycin; Sisomicin; Tobramycin
sul1_5	1–867/867	99.89	EU780013	sul1	Sulfamethoxazole
aadA2_1	18–819/819	99.88	NC_010870	aadA2	Spectinomycin; Streptomycin
dfrA12_8	1–498/498	100	AM040708	dfrA12	Trimethoprim
FloR_4	1–1215/1215	99.83	CP136914	FloR	Chloramphenicol; Florfenicol
tet(A)_6	1–1275/1275	99.92	AF534183	tet(A)	Doxycycline; Tetracycline
aph(6)-Id_1	1–837/837	99.88	M28829	aph(6)-Id	Streptomycin
aph(3**″**)-Ib_5	1–804/804	100	AF321551	aph(3″)-Ib	Streptomycin
sul2_2	1–816/816	100	AY034138	sul2	Sulfamethoxazole
OqxA_1	1–1176/1176	100	EU370913	OqxA	Chloramphenicol; Ciprofloxacin; Nalidixic_acid; Trimethoprim
OqxB_1	1–3153/3153	100	EU370913	OqxB	**Chloramphenicol; Ciprofloxacin;** Nalidixic acid; Trimethoprim
mph(A)_2	1–921/921	99.67	U36578	mph(A)	Azithromycin; Erythromycin; Spiramycin; Telithromycin

**Table 3:** Fractional inhibitory concentration (ΣFIC) indices from interactions between phytochemicals and reference antibiotics.

Bacteria	Phytos	AMX	PB	PG	OX	DIC	CIP	CHL	CT	CZ	ERY	GEN	TIC	TET	VAN
*B. cereus*	Gallic acid	2.50	-	-	-	-	2.44	-	-	-	2.44	3.03	-	-	3.98
	Catechin	**0.75**	-	-	-	-	2.19	-	-	-	1.13	1.25	-	-	1.50
	Naringenin	2.50	-	-	-	-	2.44	-	-	-	2.44	**0.77**	-	-	3.98
	Corilagin	**0.19**	-	-	-	-	2.44	-	-	-	2.44	1.50	-	-	2.00
*S. flexneri*	Gallic acid	1.25	-	-	-	-	-	1.50	2.50	-	-	1.50	-	1.50	-
	Ellagic acid	1.50	-	-	-	-	-	2.00	2.25	-	-	3.00	-	2.53	-
	Catechin	3.00	-	-	-	-	-	4.00	2.25	-	-	2.00	-	1.50	-
	Corilagin	2.50	-	-	-	-	-	3.00	2.50	-	-	2.00	-	1.50	-
	Quercetin	3.00	-	-	-	-	-	4.00	2.25	-	-	3.00	-	2.53	-
*S. sonnei*	Gallic acid	**0.75**	-	-	-	-	-	**0.75**	2.19	**1.00**	-	**0.75**	-	1.25	-
	Ellagic acid	1.25	-	-	-	-	-	3.00	2.19	4.00	-	3.00	-	1.25	-
	Catechin	1.25	-	-	-	-	-	3.00	2.19	3.00	-	3.00	-	1.25	-
	Quercetin	1.25	-	-	-	-	-	1.50	2.19	2.00	-	**1.00**	-	1.25	-
*S. typhimurium*	Gallic acid	2.50	-	-	-	-	-	-	2.50	4.00	-	**5.00**	-	3.03	-
	Ellagic acid	1.50	-	-	-	-	-	-	2.25	4.00	-	4.00	-	2.50	-
	Catechin	1.50	-	-	-	-	-	-	**4.50**	4.00	-	3.00	-	2.53	-
	Quercetin	**5.00**	-	-	-	-	-	-	**5.00**	**6.00**	-	**5.00**	-	3.03	-
*S. aureus*	Gallic acid	**0.75**	-	**0.75**	-	-	3.98	-	**5.00**	3.98	1.50	-	-	2.25	3.00
	Ellagic acid	**0.75**	-	**0.75**	-	-	2.00	-	1.25	2.00	1.50	-	-	1.13	1.50
	Catechin	**0.75**	-	**0.19**	-	-	2.00	-	1.25	0.99	1.50	-	-	2.25	1.50
	Naringenin	**0.75**	-	1.50	-	-	2.00	-	**5.00**	2.00	1.50	-	-	1.13	1.50
	Luteolin	**0.75**	-	**0.38**	-	-	2.48	-	2.00	1.25	1.13	-	-	2.06	1.50
	Corilagin	1.06	-	1.06	-	-	2.25	-	2.06	2.25	2.52	-	-	2.00	2.13
	Quercetin	1.50	-	**0.25**	-	-	3.98	-	**5.00**	3.98	1.50	-	-	1.13	3.00
MRSA	Gallic acid	-	-	-	-	3.50	3.98	-	2.50	2.50	2.50	2.50	-	1.62	3.98
	Catechin	-	-	-	-	2.44	2.00	-	1.25	**0.62**	1.25	1.25	-	2.13	2.00
	Naringenin	-	-	-	-	1.25	**0.99**	-	1.25	1.25	1.25	1.25	-	1.06	2.00
	Luteolin	-	-	-	-	1.25	2.00	-	1.25	**0.62**	1.25	1.25	-	1.06	2.00
	Corilagin	-	-	-	-	3.00	2.25	-	2.06	1.03	2.06	2.06	-	1.50	2.25
	Quercetin	**0.25 P**	-	**0.25 P**	**0.25 P**	1.25	2.00	-	1.25	**0.62**	1.25	1.25	-	1.06	2.00
*E. coli*	Gallic acid	1.50	1.50	-	-	1.50	1.03	1.50	1.25	1.50	-	2.00	1.50	2.5	-
ESBL *E. coli*	Gallic acid	-	2.00	-	-	1.50	-	-	-	-	-	-	-	-	-
*K.* *pneumoniae*	Gallic acid	2.50	1.50	-	-	-	2.50	1.25	1.50	1.25	-	3.00	-	1.50	-
	Corilagin	-	3.00	-	-	-	2.50	4.00	1.13	4.00	-	**4.50**	-	2.25	-
ESBL *K. pneumoniae*	Gallic acid	-	-	-	-	-	2.00	-	-	-	-	-	-	-	-

The ∑FIC values of phytochemicals (Phytos) were assessed in combination with standard antibiotics against *B. cereus*, *S. flexneri*, *S. sonnei*, *S. typhimurium*, *S. aureus*, MRSA, *E. coli*, ESBL *E. coli*, and *K. pneumoniae*, and ESBL *K. pneumoniae*. **Synergistic effects (∑FIC ≤ 0.5) were shown in green**, **Additive effects (∑FIC > 0.5–1.0) in blue**, indifferent (>1.0–4.0), and **antagonistic (>4.0) in red**. **Synergistic values** with **P** means proxy MIC (2.5 µg/mL) of antibiotics used to find the interaction between quercetin and AMX (amoxicillin), PG (penicillin G), and OX (oxacillin). A dash (-) indicated inactivity of either the phytochemical or antibiotic. Reference antibiotics: AMX (amoxicillin), PB (polymyxin B), PG (penicillin G), OX (oxacillin), DIC (dicloxacillin), CIP (ciprofloxacin), CHL (chloramphenicol), CT (ceftriaxone), CZ (cefazolin), ERY (erythromycin), GEN (gentamicin), TIC (ticarcillin), TET (tetracycline), VAN (vancomycin).

**Table 4:** Minimum inhibitory concentrations (MICs) of phytochemicals (Phyto) and antibiotics (Abx) tested alone and in combination at fixed ratios (10:90 to 90:10), with corresponding fold reduction values and fractional inhibitory concentration (FIC) indices against the tested bacterial strains.

Strain	MIC Abx Alone (µg/mL)	MIC Phyto Alone (µg/mL)	Ratio (Abx:Phyto)	MIC Abx in Combo (µg/mL)	Fold MIC Reduction (Abx)	MIC Phyto in Combo (µg/mL)	Fold MIC Reduction (Phyto)	FIC	Interaction
*B. cereus*	Amoxicillin (2.5)	Corilagin (62.5)	10:90	0.02	63	14.1	4	0.24	Synergistic
			20:80	0.03	42	12.5	5	0.22	
			30:70	0.05	25	10.9	6	0.22	
			40:60	0.06	21	9.4	7	0.20	
			50:50	0.08	16	7.8	8	0.19	
			60:40	0.09	14	6.3	10	0.17	
			70:30	0.11	11	4.7	13	0.16	
			80:20	0.13	10	3.1	20	0.15	
			90:10	0.28	4	3.1	20	0.27	
*S. aureus*	Penicillin G (1.25)	Catechin (625)	10:90	0.03	42	281.3	2	0.47	Synergistic
			20:80	0.03	42	125.0	5	0.22	
			30:70	0.05	25	109.4	6	0.22	
			40:60	0.06	21	93.8	7	0.20	
			50:50	0.08	16	78.1	8	0.19	
			60:40	0.09	14	62.5	10	0.17	
			70:30	0.22	6	93.8	7	0.33	
			80:20	0.25	5	62.5	10	0.30	
			90:10	0.56	2	62.5	10	0.55	Additive
*S. aureus*	Penicillin G (1.25) Penicillin G (1.25)	Luteolin (62.5) Luteolin (62.5)	10:90	0.03	42	28.1	2	0.47	Synergistic Synergistic
			20:80	0.06	21	25.0	3	0.45	
			30:70	0.09	14	21.9	3	0.42	
			40:60	0.13	10	18.8	3	0.40	
			50:50	0.16	8	15.6	4	0.38	
			60:40	0.19	7	12.5	5	0.35	
			70:30	0.22	6	9.4	7	0.33	
			80:20	0.25	5	6.3	10	0.30	
			90:10	0.28	4	3.1	20	0.27	
*S. aureus*	Penicillin G (1.25)	Quercetin (1250)	10:90	0.06	21	562.5	2	0.50	Synergistic
			20:80	0.13	10	500.0	3	0.50	
			30:70	0.19	7	437.5	3	0.50	
			40:60	0.13	10	187.5	7	0.25	
			50:50	0.16	8	156.3	8	0.25	
			60:40	0.19	7	125.0	10	0.25	
			70:30	0.22	6	93.8	13	0.25	
			80:20	0.25	5	62.5	20	0.25	
			90:10	0.28	4	31.3	40	0.25	
MRSA	Penicillin G (2.5, proxy value)	Quercetin (2500)	10:90	0.13	19	1125.0	2	0.50	Synergistic
			20:80	0.25	10	1000.0	3	0.50	
			30:70	0.19	13	437.5	6	0.25	
			40:60	0.25	10	375.0	7	0.25	
			50:50	0.31	8	312.5	8	0.25	
			60:40	0.19	13	125.0	20	0.13	
			70:30	0.22	11	93.8	27	0.13	
			80:20	0.25	10	62.5	40	0.13	
			90:10	0.28	9	31.3	80	0.12	

The “MIC alone” values represent the baseline inhibitory concentrations of each agent when used individually. Fold reduction was calculated as the ratio of MIC alone to MIC in combination. FIC index values were interpreted as follows: ≤0.5 = synergy; >0.5–1.0 = additive. MICs in combination and fold reduction in antibiotics (Abx) and phytochemicals (Phyto) are depicted in red and green colours, respectively.

**Table 5:** List of phytochemicals was investigated in this study.

Product Code	Phytochemical	Chemical Formula	Molecular Weight (g/mol)	Purity
G7384	Gallic acid	C_7_H_6_O_5_	170.12	>97–102.5% (titration)
14668	Ellagic acid	C_14_H_6_O_8_	302.197	>98%
N5893	Naringenin	C_15_H_12_O_5_	272.25	≥95%
L9283	Luteolin	C_15_H_10_O_6_	286.24	>98% (TLC)
G0424	Corilagin	C_27_H_22_O_18_	634.5	>98%
C1251	Catechin hydrate	C_15_H_16_O_7_	308.28	≥98% (HPLC)
1351800	Isorhamnetin	C_16_ H_12_ O_7_	316.3	≥98%
49513	Vitexin	C_21_H_20_O_10_	432.38	>98%
H4125	Hesperetin	C_16_H_14_O_6_	302.28	>98%
R5143	Rutin hydrate	C_27_H_32_O_17_	628.5	>94% (HPLC)
60010	Kaempferol	C_15_H_10_O_6_	286.24	>97% (HPLC)
Q4951	Quercetin	C_15_H_10_O_7_	302.23	≥95% (HPLC)
92342	Galangin	C_15_H_10_O_5_	270.24	≥98%
00180585	Hyperoside	C_21_H_20_O_12_	464.4	>98%
SML3174	Icariside II	C_27_H_30_O_10_	514.5	>98% (HPLC)

## Data Availability

Data are either presented within the manuscript or are available from the corresponding author upon reasonable request.

## Supplementary Materials

The following supporting information can be downloaded at https://www.mdpi.com/article/10.3390/ijms27135825/s1.

## Abbreviations

The following abbreviations are used in this manuscript:

ATCC	American type culture collection
ESBL	Extended spectrum β lactamase
DMSO	Dimethyl sulfoxide
HDFa	Human Dermal Fibroblasts (adult).
FIC	Fractional inhibitory concentration
MH	Mueller Hinton
MIC	Minimum inhibitory concentration
MRSA	Methicillin resistance *S. aureus*
INT	Iodonitrotetrazolium chloride
SEM	Standard error of the mean
