# Supplementary material for: Potentiation of Penicillin G and Selected β-Lactams with Quercetin Against Multidrug-Resistant Bacteria: Mechanistic Insights, Antibacterial Phytochemicals, and Toxicity Evaluation
Source: Int J Mol Sci. 2026 Jun 27;27(13):5825. doi: 10.3390/ijms27135825 (PMC13361247; doi:10.3390/ijms27135825)
Supplement: Supplementary file 1 [file ijms-27-05825-s001.zip › ijms-4377917-supplementary.pdf]

International Journal of Molecular Sciences

Electronic Supplementary File

MDPI

Article

Potentiation of penicillin G and selected β-lactams with quercetin against multidrug-resistant bacteria: Mechanistic insights, antibacterial phytochemicals, and toxicity evaluation

Gagan Tiwana[1], Matthew J. Cheesman[1*] and Ian E. Cock[2]

[1] School of Pharmacy and Medical Sciences, Griffith University, Southport, QLD 4222, Australia; gagan.tiwana@griffithuni.edu.au (G.T.); m.cheesman@griffith.edu.au (M.J.C)
[2] School of Environment and Science, Griffith University, Brisbane, QLD 4111, Australia I.Cock@griffith.edu.au (I.E.C)
* Correspondence: m.cheesman@griffith.edu.au; Tel.: +61 755529230

Academic Editor: Firstname Last-name

Received: date
Revised: date
Accepted: date
Published: date

Copyright: © 2026 by the authors. Submitted for possible open access publication under the terms and conditions of the Creative Commons Attribution (CC BY) license.

Supplementary Table S1. Selected phytochemicals identified in the plant extracts. **TB** - *Terminalia bellirica*, **TCh** - *Terminalia chebula*, **EO** - *Phyllanthus emblica*, **PN** - *Phyllanthus niruri*, **AzI** - *Azadirachta indica*, **OT** - *Ocimum tenuiflorum*, **SN** - *Solanum nigrum*. ✓ = phytochemical present, - means phytochemical does not present in the plant extract.

S. No: & Formula		Selected Compounds	TB	TCh	EO	PN	AzI	OT	SN
1.	$C_{27}H_{30}O_{16}$	Aureusidin 4,6-diglucoside	-	-	✓	-	-	-	-
2.	$C_{21}H_{18}O_{12}$	Aureusidin 6-glucuronide	✓	✓	-	-	-	-	-
3.	$C_{21}H_{20}O_{14}$	Hibiscetin 3-glucoside	-	-	✓	-	-	-	-
4.	$C_{22}H_{20}O_{10}$	3'-O-Methylderhamnosylmaysin	-	✓	✓	-	-	-	-
5.	$C_{14}H_{6}O_{8}$	Ellagic acid	✓	✓	✓	✓	✓	-	-
6.	$C_{20}H_{16}O_{12}$	Ellagic acid 2-rhamnoside	✓	✓	✓	-	-	-	-
7.	$C_{17}H_{12}O_{8}$	3,4,3'-Tri-O-methylellagic acid	✓	-	-	-	-	-	-
8.	$C_{19}H_{14}O_{12}$	Ellagic acid arabinoside	-	-	✓	-	✓	-	-
9.	$C_{14}H_{6}O_{9}$	flavellagic acid	-	-	-	-	✓	-	-
10.	$C_{14}H_{16}O_{10}$	Theogallin	✓	-	-	-	-	-	-
11.	$C_{7}H_{6}O_{5}$	Gallic acid	✓	✓	✓	✓	✓	-	-
12.	$C_{20}H_{20}O_{14}$	Gallic acid 3-O-(6-galloylglucoside)	✓	-	-	-	-	-	-
13.	$C_{29}H_{26}O_{16}$	Isorhamnetin 3- (6''-galloylglucoside)	-	✓	-	-	-	-	-
14.	$C_{14}H_{10}O_{9}$	Digallic acid	-	-	-	-	✓	-	-
15.	$C_{15}H_{14}O_{7}$	(+) -Gallocatechin	-	-	-	-	✓	-	-
16.	$C_{15}H_{14}O_{6}$	Catechin	-	-	-	-	✓	-	-
17.	$C_{13}H_{16}O_{10}$	6-Galloylglucose	✓	✓	✓	-	✓	-	
18.	$C_{10}H_{12}O_{7}$	1-O-Galloylglycerol	✓	-	-	-	✓	-	-
19.	$C_{28}H_{26}O_{15}$	(2S) -5,7,3',4'-Tetrahydroxyflavanone 7-(6-galloylglucoside)	-	-	✓	-	-	-	-

 https://doi.org/10.3390/xxxxx

20.	$C_{27}H_{24}O_{18}$	1,3,4-Trigalloyl-β-D-glucopyranose	-	✓	✓	-	-	-	-
21.	$C_{27}H_{24}O_{18}$	1,3,6-Tri-O-galloyl-β-D-glucose	✓	✓	-	-	-	-	-
22.	$C_{27}H_{24}O_{18}$	1,2,6-Trigalloyl-β-D-glucopyranose	✓	✓	✓	-	✓	-	-
23.	$C_{27}H_{24}O_{18}$	1,4,6-Trigalloyl-β-D-glucopyranose	-	✓	-	-	-	-	-
24.	$C_{22}H_{22}O_{11}$	6-O-[(2E)-3-Phenyl-2-propenoyl]-1-O-(3,4,5-trihydroxybenzoyl)-β-D-glucopyranose	-	✓	-	-	-	-	-
25.	$C_{20}H_{20}O_{14}$	1,6-Bis-O-(3,4,5-trihydroxybenzoyl) hexopyranose	-	✓	✓	✓	-	-	-
26.	$C_{28}H_{36}O_{11}$	3,7-Dihydroxy-4,5-dimethoxy-8-prenylflavan 7-O-β-D-glucopyranoside	-	-	-	-	✓	-	-
27.	$C_{14}H_{18}O_{10}$	Methyl 6-O-galloyl-β-D-glucopyranoside	-	-	✓	-	-	-	-
28.	$C_{21}H_{22}O_{14}$	Methyl 4,6-di-O-galloyl-β-D-glucopyranoside	✓	-	✓	-	-	-	-
29.	$C_{29}H_{26}O_{15}$	6-Cinnamoyl-1,2-digalloylglucose	-	✓	✓	-	-	-	-
30.	$C_{29}H_{26}O_{15}$	2-Cinnamoyl-1,6-digalloyl-β-D-glucopyranose	-	✓	✓	-	-	-	-
31.	$C_{35}H_{30}O_{18}$	Naringenin 7- (4,6-digalloylglucoside)	-	-	-	-	-	✓	-
32.	$C_{15}H_{12}O_{5}$	Naringenin	-	✓	-	-	✓	-	-
33.	$C_{30}H_{28}O_{12}$	Naringenin 7- (2-p-Coumaroylglucoside)	-	-	✓	-	-	-	-
34.	$C_{20}H_{20}O_{9}$	Chalconaringenin 2'-xyloside	-	-	-	-	✓	-	-
35.	$C_{28}H_{24}O_{15}$	Isoorientin 2''-O-gallate	-	-	✓	-	-	-	-
36.	$C_{28}H_{24}O_{13}$	Isoorientin 2''-p-hydroxybenzoate	-	✓	-	-	-	-	-
37.	$C_{21}H_{20}O_{11}$	Orientin	-	-	-	✓	-	-	-
38.	$C_{28}H_{24}O_{14}$	2''-O-Galloylisovitexin	-	✓	-	-	-	-	-
39.	$C_{21}H_{20}O_{10}$	Vitexin	-	✓	-	✓	-	-	-
40.	$C_{8}H_{8}O_{5}$	Methyl gallate	✓	✓	✓	-	-	-	-
41.	$C_{10}H_{12}O_{5}$	Propyl gallate	-	✓	-	-		-	-
42.	$C_{23}H_{20}O_{10}$	Epicatechin 3-O- (3-O-methylgallate)	✓	-	-	-	-	-	-
43.	$C_{28}H_{28}O_{12}$	Epicatechin 5-O-β-D-glucopyranoside-3-	✓	-	-	-	-	-	-

https://doi.org/10.3390/xxxxx

		benzoate							
44.	C29 H22 O14	Epicatechin 3,5-di-O-gallate	-	-	✓	-	-	-	-
45.	C15 H14 O6	Epicatechin	-	-	-	✓	-	-	-
46.	C22 H18 O9	Epiafzelechin 3-O-gallate	-	✓	-	-	-	-	-
47.	C22 H18 O10	Robinetinidol 3-O-gallate	-	-	✓	-	-	-	-
48.	C15 H10 O6	Kaempferol	-	-	✓	✓	✓	✓	-
49.	C24 H22 O14	Kaempferol 3-O-(6-malonyl-glucoside)	-	-	-	-	✓	-	-
50.	C30 H26 O13	Kaempferol 3- (6''-p-coumarylgalactoside)	-	-	-	-	-	✓	-
51.	C21 H20 O11	Kaempferol 3-α-D-galactoside	-	-	-	-	-	-	-
52.	C15 H10 O6	Luteolin	-	-	-	-	-	✓	-
53.	C21 H18 O13	6-Hydroxyluteolin 6-glucuronide	-	-	✓	-	-	-	-
54.	C21 H18 O13	8-Hydroxyluteolin 8-glucuronide	-	-	-	-	✓	-	-
55.	C22 H22 O12	6-Methoxyluteolin 7-glucoside	-	-	✓	-	-	-	-
56.	C22 H20 O13	6-Methoxyluteolin 7-glucuronide	-	-	✓	-	-	-	-
57.	C23 H22 O13	6-Methoxyluteolin 7-glucuronide methyl ester	-	-	✓	-	-	-	-
58.	C6 H6 O3	Pyrogallol	✓	✓	✓	✓	✓	-	-
59.	C12 H14 O9	Pyrogallol-2-O-glucuronide	✓	✓	✓	-	-	-	-
60.	C15 H10 O5	Galangin	-	-	-	-	-	✓	-
61.	C15 H10 O7	Quercetin	✓	✓	✓	✓	✓	✓	✓
62.	C21 H20 O12	Quercetin-3β-D-glucoside	-	-	-	✓	✓	✓	✓
63.	C21 H18 O13	Quercetin 3-O-glucuronide	✓	-	-	-	-	-	-
64.	C27 H30 O17	Quercetin 3-glucosyl- (1->6) -galactoside	-	-	-	-	-	-	✓
65.	C23 H22 O13	Quercetin 3- (6''-ethylglucuronide)	-	-	✓	-	-	-	-
66.	C21 H18 O13	Quercetin 7-glucuronide	-	✓	-	-	-	-	-
67.	C23 H22 O13	Quercetin 3- (6''-acetylgalactoside)	-	-	-	-	✓	-	-
68.	C20 H18 O12	Quercetin 4'-galactoside	-	✓	-	-	-	-	-

https://doi.org/10.3390/xxxxx

69.	$C_{27}H_{30}O_{16}$	Quercetin 3-O-rhamnoside-7-O-glucoside	✓	-	-	✓	✓	-	-
70.	$C_{14}H_{12}O_{11}$	(+)-Chebulic acid	✓	✓	✓	-	✓	-	-
71.	$C_{30}H_{28}O_{13}$	(2S) -5,7,3',4'-Tetrahydroxyflavanone 7-(6-p-coumaroylglucoside)	-	-	✓	-	-	-	-
72.	$C_{9}H_{8}O_{3}$	2-Hydroxycinnamic acid	-	-	-	-	✓	-	-
73.	$C_{15}H_{10}O_{9}$	3,5,6,7,2',3',4'-Heptahydroxyflavone	✓	-	-	-	-	-	-
74.	$C_{17}H_{16}O_{8}$	3,5,7,3',4',5'-Hexahydroxy-6,8-dimethylflavanone	-	-	✓	-	-	-	-
75.	$C_{16}H_{12}O_{7}$	3-Methoxy-5,7,3',4'-tetrahydroxy-flavone	-	-	-	-	-	-	✓
76.	$C_{19}H_{18}O_{5}$	4',7-Dimethoxy-6,8-dimethyl-5-hydroxyflavone	✓	-	-	-	-	-	-
77.	$C_{24}H_{24}O_{13}$	5,2'-Dihydroxy-7,8,6'-trimethoxyflavone 2'-glucuronide	-	-	✓	-	-	-	-
78.	$C_{25}H_{28}O_{15}$	5,7,3',5'-Tetrahydroxy-3,6,8,4'-tetramethoxyflavone 3'-glucoside	-	-	✓	-	-	-	-
79.	$C_{18}H_{16}O_{4}$	5,7-Dimethoxy-6-C-methylflavone	-	✓	-	-	-	-	-
80.	$C_{21}H_{24}O_{6}$	5-O-Methyl-8-prenylafzelechin-4β-ol	-	-	-	-	✓	-	-
81.	$C_{22}H_{20}O_{12}$	6-O-Methylscutellarin	-	-	-	-	-	✓	-
82.	$C_{23}H_{24}O_{10}$	7-Hydroxy-5,6-dimethoxyflavone 7-glucoside	-	✓	-	-	-	-	-
83.	$C_{15}H_{14}O_{2}$	7-Hydroxyflavan	✓	✓	✓	-	-	-	-
84.	$C_{21}H_{18}O_{14}$	8-Hydroxytricetin 7-glucuronide	-	-	✓	-	-	-	-
85.	$C_{21}H_{20}O_{10}$	Afzelin	-	-	-	-	✓	-	-
86.	$C_{15}H_{24}O_{9}$	Ajugol	-	-	-	-	✓	-	-
87.	$C_{42}H_{30}O_{9}$	alpha-Viniferin	-	-	-	-	✓	-	-
88.	$C_{20}H_{22}O_{9}$	Anadanthoside	-	-	-	-	✓	-	-
89.	$C_{15}H_{10}O_{5}$	Apigenin	-	-	-	✓	-	✓	-
90.	$C_{30}H_{48}O_{6}$	Arjungenin	-	-	-	-	✓	-	-
91.	$C_{21}H_{22}O_{11}$	Astilbin	-	-	-	-	✓	-	-
92.	$C_{21}H_{20}O_{11}$	Astragalin	-	-	-	✓	-	-	-

93.	C27 H30 O10	Baohuoside 1	-	✓	-	-	-	-	-
94.	C24 H28 O13	Barbatoflavan	✓	-	-	-	-	-	-
95.	C16 H18 O9	Biflorin	-	-	-	-	✓	-	-
96.	C20 H22 O5	Brosimacutin C	✓	✓	✓	-	-	-	-
97.	C10 H12 O4	Cantharidin	-	-	-	-	✓	-	-
98.	C22 H20 O13	Carminic acid	✓	-	-	-	-	-	-
99.	C14 H28 O8	Caryophyllan	-	-	-	-	✓	-	-
100.	C27 H34 O15	Catechin 3-O-rutinoside	-	-	-	-	✓	-	-
101.	C16 H18 O9	Chlorogenic acid	-	-	-	-	-	-	✓
102.	C32 H38 O12	chrysoeriol 7-O-neohesperidoside	-	✓	-	-	-	-	-
103.	C27 H22 O18	Corilagin	-	✓	✓	-	-	-	-
104.	C26 H28 O14	Corymboside	-	-	-	✓	-	-	-
105.	C7 H12 O6	D-(-)-Quinic acid	✓	✓	-	-	✓	-	-
106.	C19 H18 O5	Eucalyptin	-	✓	-	-	-	-	-
107.	C11 H10 O4	Eugenitol	-	✓	-	-	-	-	-
108.	C10 H12 O2	Eugenol	-	-	-	-	-	✓	-
109.	C27 H32 O7	Exiguaflavanone E	-	-	-	-	✓	-	-
110.	C25 H30 O7	Exiguaflavanone M	✓	-	-	-	-	-	-
111.	C15 H10 O6	Fisetin	-	-	✓	✓	✓	✓	-
112.	C26 H30 O10	Flavaprin	✓	-	-	-	-	-	-
113.	C17 H12 N2 O4	Flazin	-	-	✓	-	-	-	-
114.	C15 H12 O6	(-)-Fustin	-	-	-	✓	-	-	-
115.	C28 H38 O2	Gymnasterone D	-	✓	-	-	-	-	-
116.	C20 H20 O14	Hamamelitannin	✓	✓	✓	-	✓	-	-
117.	C21 H22 O10	Hemiphloin	-	-	✓	-	-	-	-
118.	C16 H14 O6	Hesperetin	-	-	-	-	-	✓	-

https://doi.org/10.3390/xxxxx

119.	C24 H28 O7	Heteroflavanone B	✓	-	-	-	✓	-	-
120.	C21 H20 O12	Hyperoside	-	-	-	-	✓	-	-
121.	C33 H40 O15	Icariin	-	✓	-	-	✓	-	-
122.	C27 H32 O11	Icaritin 3-rhamnoside	-	-	-	-	✓	-	-
123.	C26 H28 O10	Ikarisoside A	✓	-	-	-	-	-	-
124.	C16 H28 O2	Isoambrettolide	-	✓	-	-	-	-	-
125.	C11 H10 O5	Isofraxidin	-	-	-	-	✓	-	-
126.	C15 H12 O4	Isoliquiritigenin	-	-	✓	-	-	-	-
127.	C17 H16 O4	Isoliquiritigenin 4,4'-dimethyl ether	-	-	-	-	✓	-	-
128.	C39 H62 O13	Isonuatigenin 3-[rhamnosyl-(1->2)-glucoside]	-	✓	-	-	-	-	-
129.	C16 H12 O7	Isorhamnetin	-	-	-	-	✓	-	-
130.	C26 H32 O8	Kushenol H	-	-	-	-	✓	-	-
131.	C19 H22 O7	Machaerol B	-	-	-	-	✓	-	-
132.	C21 H20 O11	Maritimein	✓	-	-	-	-	-	-
133.	C21 H18 O13	Miquelianin	✓	✓	-	✓	-	-	✓
134.	C21 H20 O13	myricetin 3-O-β-D-galactopyranoside	-	-	-	-	✓	-	-
135.	C21 H20 O12	Myricitrin	✓	-	✓	-	✓	-	-
136.	C14 H6 O6	Nasutin A	-	-	-	-	-	✓	-
137.	C28 H34 O15	Neohesperidin	✓	-	-	-	-	✓	-
138.	C27 H30 O15	Nictoflorin	-	-	-	-	✓	-	-
139.	C12 H16 O8	Phlorin	✓	✓	-	-	-	-	-
140.	C29 H32 O10	Pilosanol A	✓	-	-	-	-	-	-
141.	C17 H18 O6	Protofarrerol	-	-	-	-	✓	-	-
142.	C27 H30 O16	Rutin	✓	✓	-	✓	✓	✓	✓
143.	C27 H22 O18	Sanguiin H4	✓	✓	✓	-	-	-	-
144.	C21 H10 O13	Sanguisorbic acid dilactone	✓	✓	-	-	-	-	-

https://doi.org/10.3390/xxxxx

145.	C20 H20 O8	Sigmoidin G	-	✓	-	-	-	-	-
146.	C25 H22 O9	Silandrin	-	-	-	-	-	✓	-
147.	C20 H20 O7	Tangeretin	✓	-	-	-	-	-	-
148.	C15 H12 O7	(2R,3R) -Taxifolin	-	-	-	-	✓	-	-
149.	C15 H12 O7	Taxifolin	-	-	-	-	✓	-	-
150.	C21 H20 O13	Telephioidin	-	-	✓	-	-	-	-
151.	C21 H20 O8	Torosaflavone A	-	-	✓	-	-	-	-
152.	C21 H20 O11	Trifolin	✓	-	✓	✓	✓	✓	-
153.	C7 H7 N O2	Trigonelline	-	✓	-	-	✓	✓	✓
154.	C9 H6 O4	Aesculetin	-	-	-	-	✓	-	-
155.	C20 H30 O12	Bioside	-	-	-	-	✓	-	-
156.	C9 H8 O7 S	Caffeic acid 3-O-sulfate	-	-	-	-	✓	-	-
157.	C13 H12 O9	Caftaric acid	-	-	-	-	✓	-	-
158.	C6 H6 O2	Catechol	-	-	-	-	✓	-	-
159.	C20 H16 O5	Ciliatin A	-	✓	-	-		-	-
160.	C29 H42 O9	Corchoroside A	-	-	-	-	✓	-	-
161.	C17 H24 O10	Geniposide	-	-	-	-	✓	-	-
162.	C27 H32 O15	Hovetrichoside D	-	-	-	-	✓	-	-
163.	C9 H7 N O	8-Hydroxyquinoline	-	-	-	-	✓	-	-
164.	C10 H7 N O3	Kynurenic acid	-	-	-	-	✓	-	-
165.	C28 H38 O13	(7'R)-(+)-Lyoniresinol 9'-glucoside	-	-	-	-	✓	-	-
166.	C26 H36 O11	Mascaroside	-	-	-	-	✓	-	-
167.	C16 H18 O9	Neochlorogenic acid	-	-	-	-	✓	-	✓
168.	C27 H30 O7	Nimbolide	-	-	-	-	✓	-	-
169.	C7 H6 O4	Protocatechuic acid	-	-	-	-	✓	-	-
170.	C11 H12 O5	Sinapinic acid	-	-	-	-	✓	-	-

171.	$C_{36}H_{56}O_{12}$	Tenuifolin	-	-	-	-	✓	-	-
172.	$C_{20}H_{26}O_{13}$	trans-Caffeic acid [apiosyl-(1->6)-glucosyl] ester	-	-	-	-	✓	-	-
173.	$C_{15}H_{18}O_{8}$	trans-p-Coumaric acid 4-glucoside	-	-	-	-	✓	-	-
174.	$C_{18}H_{26}O_{5}$	α-Zearalanol	-	-	-	✓	-	-	-

https://doi.org/10.3390/xxxxx

Supplementary Table S2. Antimicrobial resistance analysis of the ESBL *Escherichia coli* species using CARD sequence identification.

GENE	COVERAGE	%IDENTITY	ACCESSION	PRODUCT
CTX-M-64	1-876/876	100	AB284167.2:225-1101	CTX-M-64 is a β-lactamase.
mdtM	1-1233/1233	95.7	U00096.3:4568519-4567286	Multidrug resistance protein MdtM.
*Escherichi coli*_acrA	1-1194/1194	99.08	U00096.3:485619-484425	AcrA is a subunit of the AcrAB-TolC multidrug efflux system found in *E. coli*.
acrB	1-3150/3150	99.08	U00096.3:484403-481253	Protein subunit of AcrA-AcrB-TolC multidrug efflux complex. AcrB functions as a herterotrimer which forms the inner membrane component and is primarily responsible for substrate recognition and energy transduction by acting as a drug/proton antiporter.
AcrF	1-3105/3105	97.87	U00096.1:3415032-3418137	AcrF is a inner membrane transporter similar to AcrB.
AcrE	1-1158/1158	98.62	U00096.1:3413863-3415021	AcrE is a membrane fusion protein similar to AcrA.
AcrS	1-663/663	97.59	U00096.1:3413465-3412802	AcrS is a repressor of the AcrAB efflux complex and is associated with the expression of AcrEF. AcrS is believed to regulate a switch between AcrAB and AcrEF efflux.
bacA	1-820/822	96.58	U00096.3:3204131-3203309	The bacA gene product (BacA) recycles undecaprenyl pyrophosphate during cell wall biosynthesis which confers resistance to bacitracin.
TolC	1-1488/1488	100	FJ768952.1:0-1488	TolC is a protein subunit of many multidrug efflux complexes in Gram negative bacteria. It is an outer membrane efflux protein and is constitutively open. Regulation of efflux activity is often at its periplasmic entrance by other components of the efflux complex.
mdtA	1-1248/1248	96.23	U00096.1:2154015-2155263	MdtA is the membrane fusion protein of the multidrug efflux complex mdtABC.
mdtB	1-3123/3123	95.93	U00096.1:2155262-2158385	MdtB is a transporter that forms a heteromultimer complex with MdtC to form a multidrug transporter. MdtBC is part of the MdtABC-TolC efflux complex.
mdtC	1-3078/3078	95.58	U00096.1:2158385-2161463	MdtC is a transporter that forms a hetero-multimer complex with MdtB to form a multidrug transporter. MdtBC is part of the MdtABC-TolC efflux complex. In the absence of MdtB MdtC can form a homomultimer complex that results in a functioning efflux complex with a narrower drug specificity. mdtC corresponds to 3 loci in *Pseudomonas aeruginosa* PAO1 (gene name: muxC/muxB) and 3 loci in *Pseudomonas aeruginosa* LESB58.

cpxA	1-1374/1374	98.84	BA000007.3:4905062-4903688	CpxA is a membrane-localized sensor kinase that is activated by envelope stress. It starts a kinase cascade that activates CpxR which promotes efflux complex expression.
leuO	1-945/945	97.04	LR730402.1:740042-740987	leuO a LysR family transcription factor exists in a wide variety of bacteria of the family Enterobacteriaceae and is involved in the regulation of unidentified genes affecting the stress response and pathogenesis expression. LeuO is also an activator of the MdtNOP efflux pump.
ugd	1-1167/1167	98.11	U00096.1:2099613-2098446	PmrE is required for the synthesis and transfer of 4-amino-4-deoxy-L-arabinose (Ara4N) to Lipid A which allows gram-negative bacteria to resist the antimicrobial activity of cationic antimicrobial peptides and antibiotics such as polymyxin.
emrB	1-1539/1539	98.44	U00096.1:2812615-2814154	emrB is a translocase in the emrB -TolC efflux protein in *E. coli*. It recognizes substrates including carbonyl cyanide m-chlorophenylhydrazone (CCCP) nalidixic acid and thioloactomycin.
emrA	1-1173/1173	98.3	AP009048.1:2810082-2811255	EmrA is a membrane fusion protein providing an efflux pathway with EmrB and TolC between the inner and outer membranes of *E. coli* a Gram-negative bacterium.
emrR	1-531/531	99.44	U00096.3:2810769-2811300	EmrR is a negative regulator for the EmrAB-TolC multidrug efflux pump in *E. coli.* Mutations lead to EmrAB-TolC overexpression.
*Escherichia*_ *coli*_mdfA	1-1233/1233	96.76	JQ394987.1:0-1233	Multidrug efflux pump in *E. coli*. This multidrug efflux system was originally identified as the Cmr/CmlA chloramphenicol exporter.
marA	1-384/384	99.74	AP009048.1:1621287-1621671	In the presence of antibiotic stress *E. coli* overexpresses the global activator protein MarA which besides inducing MDR efflux pump AcrAB also down-regulates synthesis of the porin OmpF.
evgS	1-3594/3594	96.72	U00096.1:2484373-2487967	EvgS is a sensor protein that phosphorylates the regulatory protein EvgA. evgS corresponds to 1 locus in *Pseudomonas aeruginosa* PAO1 and 1 locus in Pseudomonas aeruginosa LESB58.
evgA	1-615/615	99.19	BA000007.3:3212025-3212640	EvgA when phosphorylated is a positive regulator for efflux protein complexes emrKY and mdtEF. While usually phosphorylated in a EvgS dependent manner it can be phosphorylated in the absence of EvgS when overexpressed.

emrK	1-1056/1056	97.25	D78168.1:536-1592	emrK is a membrane fusion protein that is a homolog of EmrA. Together with the inner membrane transporter EmrY and the outer membrane channel TolC it mediates multidrug efflux.
emrY	1-1539/1539	98.05	D78168.1:1591-3130	emrY is a multidrug transport that moves substrates across the inner membrane of the Gram-negative *E. coli*. It is a homolog of emrB.
PmrF	1-969/969	98.04	U00096.1:2367070-2368039	PmrF is required for the synthesis and transfer of 4-amino-4-deoxy-L-arabinose (Ara4N) to Lipid A which allows gram-negative bacteria to resist the antimicrobial activity of cationic antimicrobial peptides and antibiotics such as polymyxin. pmrF corresponds to 1 locus in *Pseudomonas aeruginosa* PAO1 and 1 locus in *Pseudomonas aeruginosa* LESB58.
YojI	1-1644/1644	97.32	U00096.3:2308615-2306971	YojI mediates resistance to the peptide antibiotic microcin J25 when it is expressed from a multicopy vector. YojI is capable of pumping out microcin molecules. The outer membrane protein TolC in addition to YojI is required for export of microcin J25 out of the cell. Microcin J25 is thus the first known substrate for YojI.
H-NS	1-414/414	99.28	BA000007.3:1738104-1737690	H-NS is a histone-like protein involved in global gene regulation in Gram-negative bacteria. It is a repressor of the membrane fusion protein genes acrE mdtE and emrK as well as nearby genes of many RND-type multidrug exporters.
APH(3')-IIa	1-795/795	100	V00618.1:150-945	APH(3')-IIa is a transposon-encoded aminoglycoside phosphotransferase in *E. coli.*
msbA	1-1749/1749	99.03	U00096.3:966620-968369	MsbA is a multidrug resistance transporter homolog from *E. coli* and belongs to a superfamily of transporters that contain an adenosine triphosphate (ATP) binding cassette (ABC) which is also called a nucleotide-binding domain (NBD). MsbA is a member of the MDR-ABC transporter group by sequence homology. MsbA transports lipid A a major component of the bacterial outer cell membrane and is the only bacterial ABC transporter that is essential for cell viability.
OmpA	18-1125/1125	84.21	FO834906.1:2175099-2173974	OmpA is a porin that confers resistance to β-lactam antibiotics.

mdtG	1-1227/1227	98.21	CP000800.1:1192954-1191727	The MdtG protein also named YceE appears to be a member of the major facilitator superfamily of transporters and it has been reported when overexpressed to increase fosfomycin and deoxycholate resistances. mdtG is a member of the marA-soxS-rob regulon.
mdtH	1-1209/1209	97.77	U00096.1:1125326-1124117	Multidrug resistance protein MdtH.
TEM-1	1-861/861	99.88	AL513383.1:161910-162771	TEM-1 is a broad-spectrum beta-lactamase found in many Gram-negative bacteria. Confers resistance to penicillins and first generation cephalosphorins.
rmtB	1-756/756	100	AM886293.1:116479-117235	RmtB is a 16S rRNA methyltransferase that targets mature or nearly mature 30S subunits. It transfers a methyl group from S-adenosyl-L-methionine to N7-G1405 of the 16S rRNA an aminoglycoside binding site.
sul1	1-840/840	100	JF969163.1:1053-1893	Sul1 is a sulfonamide resistant dihydropteroate synthase of Gram-negative bacteria. It is linked to other resistance genes of class 1 integrons.
qacEdelta1	1-348/348	100	U49101.1:1490-1838	QacEdelta1 is a resistance gene conferring resistance to antiseptics. It is different from QacE only at the 3'-terminus.
aadA2	1-780/780	99.87	AF156486.1:5012-5792	aadA2 is an aminoglycoside nucleotidyltransferase gene encoded by plasmids and integrons in *K. pneumoniae Salmonella* spp., *Corynebacterium glutamicum*, *C. freundii* and *Aeromonas* spp.
dfrA12	1-498/498	100	GU585907.1:22103-21605	dfrA12 is an integron-encoded dihydrofolate reductase found in *Vibrio cholerae*.
floR	1-1215/1215	99.75	AF231986.2:3307-4522	floR is a plasmid or chromosome-encoded chloramphenicol exporter that is found in *Bordetella bronchiseptica Escherichia coli Klebsiella pneumoniae Salmonella enterica* subsp. *enterica* serovar *Typhimurium* str. DT104 and *Vibrio cholerae*.
tet(A)	1-1275/1275	99.92	AF534183.1:2970-4245	TetA is a tetracycline efflux pump found in many species of Gram-negative bacteria.
APH(6)-Id	1-837/837	99.76	AF024602.1:3155-3992	APH(6)-Id is an aminoglycoside phosphotransferase encoded by plasmids integrative conjugative elements and chromosomal genomic islands in *K. pneumoniae Salmonella spp. E. coli Shigella flexneri Providencia alcalifaciens Pseudomonas spp. V. cholerae Edwardsiella tarda Pasteurella multocida* and *Aeromonas bestiarum.*

APH(3'')-Ib	1-804/804	99.75	AF313472.2:15593-16397	APH(3'')-Ib is an aminoglycoside phosphotransferase encoded by plasmids transposons integrative conjugative elements and chromosomes in Enterobacteriaceae and *Pseudomonas* spp.
sul2	1-816/816	100	AY055428.1:21084-20268	Sul2 is a sulfonamide resistant dihydropteroate synthase of Gram-negative bacteria usually found on small plasmids.
kdpE	1-678/678	97.05	U00096.3:721733-721055	kdpE is a transcriptional activator that is part of the two-component system KdpD/KdpE that is studied for its regulatory role in potassium transport and has been identified as an adaptive regulator involved in the virulence and intracellular survival of pathogenic bacteria. kdpE regulates a range of virulence loci through direct promoter binding.
Escherichia_coli_emrE	1-333/333	99.7	Z11877.1:485-818	Member of the small MDR (multidrug resistance) family of transporters; in *E. coli* this protein provides resistance against a number of positively charged compounds including ethidium bromide and erythromycin; proton-dependent secondary transporter which exchanges protons for compound translocation.
oqxA	1-1176/1176	100	EU370913.1:46651-47827	RND efflux pump conferring resistance to fluoroquinolone.
oqxB	1-3153/3153	100	EU370913.1:47850-51003	RND efflux pump conferring resistance to fluoroquinolone.
Mrx	1-1239/1239	100	AY522923.1:8983-7744	Mrx is part of the macrolide inactivation gene cluster in *Aeromonas hydrophila*.
mphA	1-906/906	100	D16251.1:2531-1625	The mphA gene encodes for resistance enzyme MPH(2')-I which preferentially inactivate 14-membered macrolides (e.g.erythromycin telithromycin roxithromycin) over 16-membered macrolides (e.g.tylosin spiramycin). It phosphorylates macrolides at 2'-OH hydroxyl of desosamine sugar of macrolides in a GTP-dependent manner.
acrD	1-3114/3114	98.68	AP009048.1:2586250-2589364	AcrD is an aminoglycoside efflux pump expressed in *E. coli*. Its expression can be induced by indole and is regulated by baeRS and cpxAR.
gadX	95-825/825	93.43	AP009048.1:3974604-3975429	GadX is an AraC-family regulator that promotes mdtEF expression to confer multidrug resistance.
gadW	1-729/729	96.02	CP015085.1:2552440-2551711	GadW is an AraC-family regulator that promotes mdtEF expression to

				confer multidrug resistance. GadW inhibits GadX-dependent activation. GadW clearly represses gadX and in situations where GadX is missing activates gadA and gadBC.
mdtF	1-3114/3114	98.04	U00096.1:3660413-3663527	MdtF is the multidrug inner membrane transporter for the MdtEF-TolC efflux complex.
mdtE	1-1158/1158	98.45	AP009048.1:3981183-3980025	MdtE is the membrane fusion protein of the MdtEF multidrug efflux complex. It shares 70% sequence similarity with AcrA.
CRP	1-633/633	99.21	AP009048.1:4154296-4153663	CRP is a global regulator that represses MdtEF multidrug efflux pump expression.
mdtP	1-1467/1467	96.66	AP009048.1:4304509-4303042	Multidrug resistance efflux pump. Could be involved in resistance to puromycin acriflavine and tetraphenylarsonium chloride.
mdtO	1-2052/2052	96.39	AP009048.1:4306557-4304505	Multidrug resistance efflux pump. Could be involved in resistance to puromycin acriflavine and tetraphenylarsonium chloride.
mdtN	1-1032/1032	97.77	AP009048.1:4307588-4306556	Multidrug resistance efflux pump. Could be involved in resistance to puromycin acriflavine and tetraphenylarsonium chloride.
eptA	1-1644/1644	97.32	AP009048.1:4340268-4338624	PmrC mediates the modification of lipid A by the addition of 4-amino-4-deoxy-L-arabinose (L-Ara4N) and phosphoethanolamine resulting in a less-negative cell membrane and decreased binding of polymyxin B.
EC-8	1-1134/1134	98.33	NG_049086.1:100-1234	EC-8 is an Extended-spectrum β-lactamase.
